# MicroRNA-mRNA Interactions at Low Levels of Compressive Solid Stress Implicate mir-548 in Increased Glioblastoma Cell Motility

**DOI:** 10.1038/s41598-019-56983-x

**Published:** 2020-01-15

**Authors:** Mark A. Calhoun, Yixiao Cui, Eileen E. Elliott, Xiaokui Mo, Jose J. Otero, Jessica O. Winter

**Affiliations:** 1 Department of Biomedical Engineering, The Ohio State University, Columbus, OH USA; 2 William G. Lowrie Department of Chemical and Biomolecular Engineering, The Ohio State University, Columbus, OH USA; 3 Department of Pathology, The Ohio State University, Columbus, OH USA; 4 Center for Biostatistics and Bioinformatics, The Ohio State University, Columbus, OH USA

**Keywords:** Cancer microenvironment, Cancer genetics

## Abstract

Glioblastoma (GBM) is an astrocytic brain tumor with median survival times of <15 months, primarily as a result of high infiltrative potential and development of resistance to therapy (i.e., surgical resection, chemoradiotherapy). A prominent feature of the GBM microenvironment is compressive solid stress (CSS) caused by uninhibited tumor growth within the confined skull. Here, we utilized a mechanical compression model to apply CSS (<115 Pa) to well-characterized LN229 and U251 GBM cell lines and measured their motility, morphology, and transcriptomic response. Whereas both cell lines displayed a peak in migration at 23 Pa, cells displayed differential response to CSS with either minimal (i.e., U251) or large changes in motility (i.e., LN229). Increased migration of LN229 cells was also correlated to increased cell elongation. These changes were tied to epigenetic signaling associated with increased migration and decreases in proliferation predicted via Ingenuity® Pathway Analysis (IPA), characteristics associated with tumor aggressiveness. miRNA-mRNA interaction analysis revealed strong influence of the miR548 family (i.e., mir-548aj, mir-548az, mir-548t) on differential signaling induced by CSS, suggesting potential targets for pharmaceutical intervention that may improve patient outcomes.

## Introduction

Glioblastoma (GBM) is a highly lethal brain tumor characterized by diffuse margins and with median survival times of ~20 months from diagnosis^1^. The diffuse invasion of GBM cells into the brain parenchyma renders curative surgical resection and radiotherapy improbable^2^. Further complicating treatment, migratory cells are less proliferative^3^ allowing them to evade drugs that target proliferating cells, i.e. temozolomide (TMZ), which is the first line of defense in GBM. Thus, recurrence is almost inevitable, generally occurring ~9 months post-resection^4^. In up to 90% of cases, recurrence occurs within a 2 cm margin of the tumor cavity^5^, implicating highly motile GBM cells that have invaded the surrounding brain parenchyma. These cells are most commonly found occupying perivascular and perineural spaces^6^. The perivascular niche presents unique challenges, as it co-opts host vasculature to alter the brain microenvironment^7^. These microenvironmental changes, among others, are part of the reason GBM has a high rate of mortality.

An important component of the GBM tumor microenvironment includes physical forces caused directly or indirectly by tumor progression. Tumor co-option of vasculature leads to leaky blood vessels^8^, blood stasis^9^, and elevated interstitial fluid pressure in the tumour^10^. In addition, tumor growth within the steric hindrance of the skull causes mechanical stresses within brain tissue, including compressive solid stress (CSS) in the radial direction and tension in the tangential direction. This suite of physical stimuli has implications in tumor pathophysiology^11^. Whereas tangential forces are most likely stored in large elements, such as axons and white matter tracts, CSS most likely acts on normal and cancerous cells. Noticeably, CSS has been shown to compress and displace vasculature surrounding the GBM tumour^12^. In breast cancer cell models, CSS enhances elongation and migration^13,14^, decreases proliferation, and increases apoptosis^15,16^. There are comparatively fewer studies in GBM, but mechanical compression has been shown to increase expression of VEGF, collagen IV and VI, and the collagen crosslinker lysyl oxidase^17^. Thus, CSS in GBM may be correlated to angiogenesis and vascular co-option; however, these results were based on high levels of CSS (physiological maximum of ~0.21 kPa in a mouse model^18^ vs. 1 kPa in that study). Increased understanding of GBM mechanobiology, specifically as it relates to CSS, is needed to elucidate how physiologically-relevant mechanical cues are translated to GBM phenotypes relating to progression (e.g., migratory, proliferatory, and with differential genetic regulation).

CSS in GBM is associated with the midline shift, a radiological hallmark in which the tumor-affected hemisphere bulges into the contralateral hemisphere. Maximum CSS occurs at the GBM tumor boundary and decreases radially in magnitude throughout the parenchyma^18^. The 2–3 cm region beyond the tumor periphery presents a critical tumor microenvironment niche in GBM, with physical stimuli acting on invasive tumor cells that escape conventional therapy (i.e., surgical resection, chemoradiotherapy). CSS decay rapidly in a radial fashion with distance from tumour (i.e., from ~100 Pa to 10 Pa in 100 µm^12^), and CSS in infiltrative tumour decays more rapidly than that in nodular tumours^12^. Studies using low CSS, closer to physiological values, would better recapitulate the mechanical environment in this critical 2 cm radius of recurrence. Such an approach could lead to novel chemotherapeutic targets that intercept mechanical cues from the GBM microenvironment. At present, it is not well understood how GBM cell signal transduction pathways are altered in response to physical stimuli. Understanding differential regulation in the context of physical stimuli is a first step to potential pharmacological intervention and by extension, better patient outcomes.

Here, we sought to elucidate the effects of CSS on the cellular and molecular level on isolated GBM cells, focusing on the microenvironment niche within 2 cm of the tumor periphery. Thus, low levels of CSS (i.e., <0.210 kPa) were employed. *In vivo*, GBM cells migrate either collectively, guided by high Rac1 and Cdc42 activity “leader” cells^6^, or as single invasive cells reciprocally interacting with host vasculature^19^. To mimic the collective cell migration scenario in the perivascular and perineural spaces, we employed a wound healing assay combined with an established CSS model that applies stress in 1D^13^, similar to radial compression forces experienced by GBM cells. This model was previously employed to study collective cell migration and cytoskeletal dynamics, and we extended this model to study both collective and single cell motility paradigms in GBM^20^. Whereas no *in vitro* model can fully mimic physiological conditions, this model facilitates access to fresh media and oxygen, decoupling CSS from other co-morbid cues in the tumor microenvironment, such as elevated interstitial fluid pressure, vascular compression, and hypoxia. In this model, we investigated migration of LN229 and U251 cells, established GBM cell lines with defined properties that permit examination of concordance with the literature. We also investigated the role of differential epigenetic signaling and predicted pathway activation using a microarray and subsequent miRNA-mRNA interaction analysis. These results suggest potential methods to mine pharmacological targets from differential signaling induced by tumor-initiated physical forces.

## Results

### Migration speed was enhanced by low CSS but decreased by high CSS

Tumor cells migrating at the tumor periphery and into the brain parenchyma persist after surgery and chemoradiation, presumably leading to recurrence. Thus, we constrained our experiments to levels of CSS reflective of the 2 cm radius of recurrence, with forces applied in 1D, similar to radial compression forces experienced by GBM cells. CSS peaks at the tumor periphery and decreases throughout this region^18^. In a mouse model, CSS was measured to a maximum of 210 Pa^18^, so we constrained our range of interest from 0 to 115 Pa (i.e., roughly half of the maximum). Pressure was applied using a modified version of a model previously used to study the leader cell migration phenotype in breast tumor cells, for which physiologically relevant CSS is much higher (i.e., ~800 Pa)^13^. In this model, cells were grown on a Transwell© insert, which facilitated access to media and prevented hypoxia. We modified this model by including a variable weight stack (Supplementary Fig. 1A) and tested the effect of CSS on GBM migration compared to controls in a wound healing assay with a gap of 500 µm over a period of 18 hr (Supplementary Fig. 1B,C).

**Figure 1:**
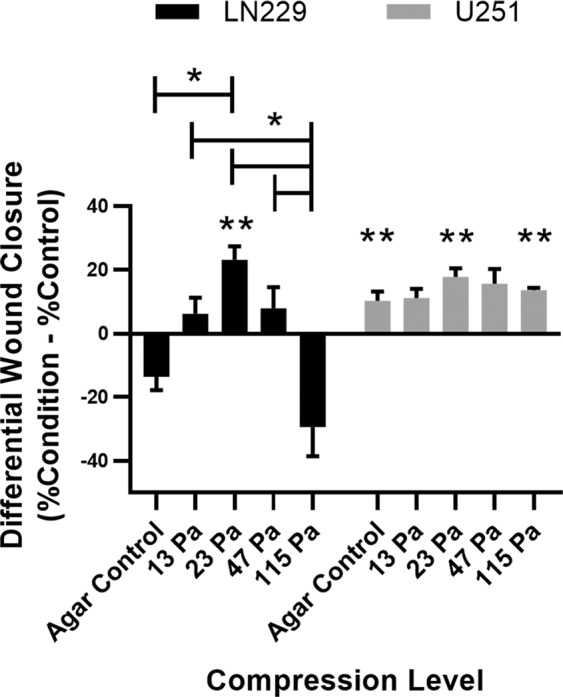
Collective cell migration reaches a maximum at 23 Pa CSS in LN229 and U251 cells. Differential wound closure: the difference of each compression level (agar control, 13 Pa, 23 Pa, 47 Pa, and 115 Pa) from its corresponding experimental control. Levels connected by a star (*) are statistically significant at α = 0.05. Conditions marked with two stars (**) are statistically significant compared to their control for each cell type at α = 0.01 after Bonferroni correction.

The no pressure (i.e., no CSS, no agar cushion) and agar (i.e., no CSS) controls did not demonstrate a statistically significant difference in wound closure in LN229, but did have a statistical difference for U251 cell lines (Fig. 1), indicating that the agar cushion alone could influence migration in a detectable manner. LN229 cells migrated faster than U251 cells, as control LN229 cells closed 57.0 ± 3.3% of the gap, whereas control U251 cells closed only 36.7 ± 3.0% of the gap. For LN229 cells at 23 Pa, the maximum migration rate observed, wound closure was significantly faster than the control, with 23.2 ± 4.3% more gap closure over 18 hr, equivalent to a ~1.4x increase (p = 0.0062). U251 cells also had a statistically significant peak in wound closure at 23 Pa, closing 17.8 ± 4.6% more of the gap than the control (p = 0.0006), a ~1.5x increase. At the highest CSS investigated of 115 Pa, LN229 cells exhibited negative wound closure compared to the control, whereas U251 cells closed 13.6 ± 5.3% more of the gap than the control (p = 0.0017). Thus, U251 cells had a positive differential wound closure at all levels of CSS. This data extends previous findings of increased cell migration under CSS to GBM cancers. Additionally, it demonstrates two migratory responses to CSS: a dramatic response in LN229 cells and a minimal response in U251 cells.

### Two cell morphology populations were observed

**Figure 2:**
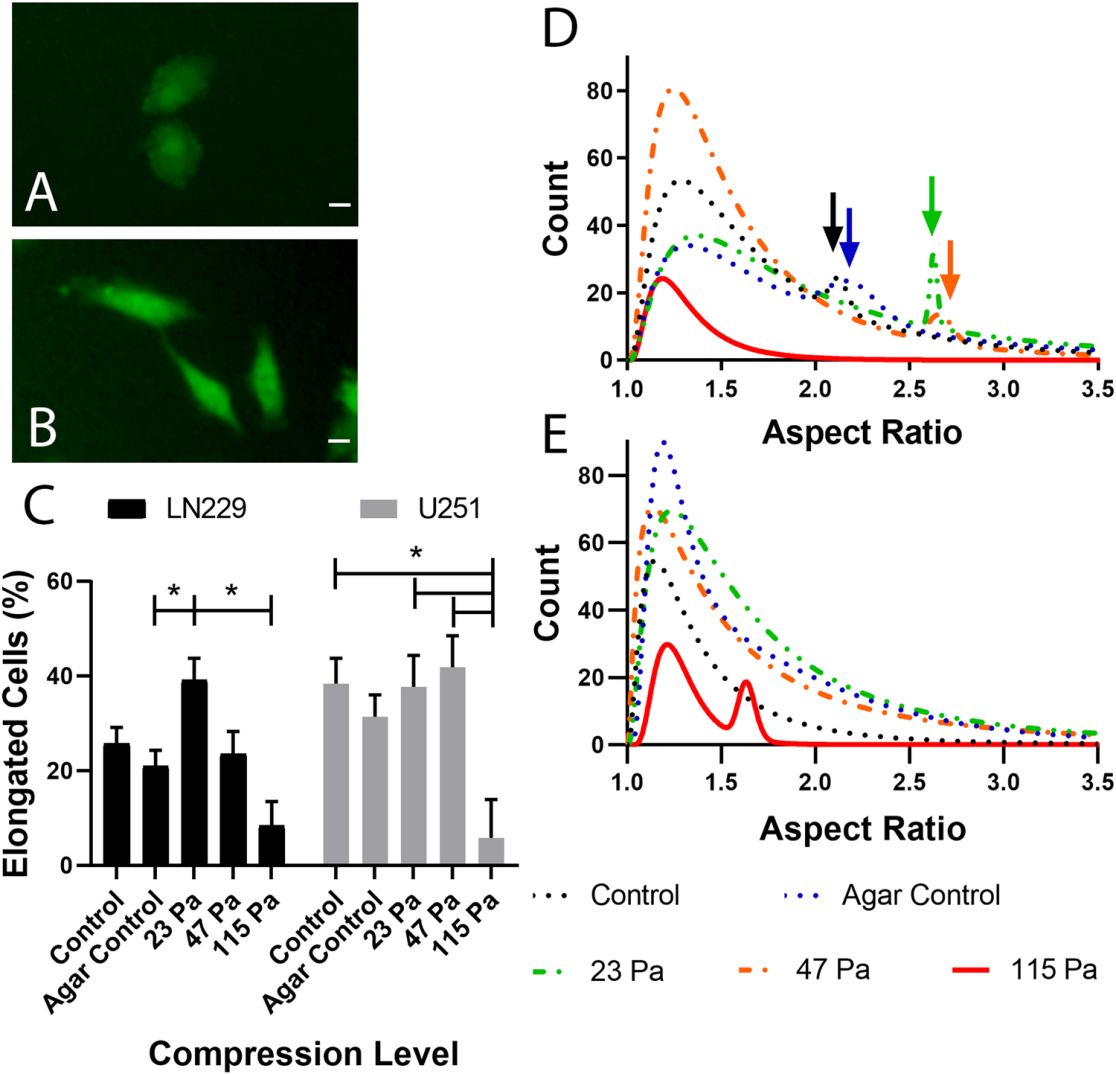
CSS induces an elongated subtype in single LN229 cells under CSS. Representative images of LN229 cells show rounded morphology in the agar control (**A**) and elongated cells at 23 Pa (**B**). Scale bar = 20 µm. (**C**) Percent elongated cells (AR ≥ 2) for LN229 and U251 cells. Levels connected by a star (*) are statistically significant at α = 0.05. Probability density functions of LN229 (**D**) and U251 (**E**) cell aspect ratio (AR) at increasing levels of CSS. Arrows indicate the second population peaks.

Next, we investigated the influence of CSS on cell morphology, which is closely related to many cell processes, including adhesion, contractility, and migration^21^. To recapitulate GBM migration through the brain parenchyma, cells were grown in a non-confluent monolayer that permitted observation of single cell morphology (Fig. 2). Qualitatively, a mixed population of rounded (Fig. 2A) and elongated cells (Fig. 2B) were present across all conditions, though their proportions varied. To quantify morphology, aspect ratio (AR), the ratio of length to width for which AR = 1 is a perfect circle and higher values (AR ≥2) indicate more elongated cells, was employed. Corresponding cell area was also assessed (Supplementary Fig. 2). For LN229 cells, the percent elongated cells was significantly higher at 23 Pa compared to the agar control and the highest CSS employed of 115 Pa (p = 0.0243, 0.0007, respectively) (Fig. 2C). At 23 Pa, 39.1 ± 4.4% of cells were elongated compared to only 21.0 ± 3.9%, or roughly half that amount, in the agar control. Conversely, U251 cells maintained a relatively high percent elongated cells across pressures <115 Pa. On average, U251 cells were 33.0 ± 3.0% elongated, whereas LN229 cells were 23.8 ± 2.2% elongated. Projected cell area results (Supplementary Fig. 2) were inversely proportional to those of AR, consistent with the assumption of incompressibility (i.e., constant volume). Cells generally had lower cell area at intermediate conditions, when AR was higher, and the largest cell areas at the 115 Pa when most cells were rounded. Morphology results mirrored those of migration, although U251 cells had significantly lower percent elongated cells at 115 Pa (5.8 ± 1.9%, p = 0.0343), which was more pronounced than declines in migration.

GBM cell elongation was further analyzed using AR histograms for each CSS level that were fit with 1- or 2-term probability density functions (PDFs) to statistically test for the presence of a second population (Fig. 2D,E). The fit for each lognormal curve was excellent (R^2^ ≥ 0.91). LN229 cells displayed a second cell population with higher AR in all conditions except 115 Pa (Fig. 2D). LN229 control and agar samples had relatively small second AR populations with peaks at 2.12 and 2.19, respectively (Table 1). At 23 and 47 Pa, these peaks shifted to higher AR with maxima at 2.63 and 2.66, respectively. Additionally, the 23 Pa secondary AR peak was relatively larger than the others, at roughly the same height as its primary peak. The secondary peak of 23 Pa was also narrower than that of 47 Pa (variance σ^2^ of 0.0001 vs 0.0015). (Supplementary Table 1). U251 cell response differed from that of LN229 cells (Fig. 2E). Only the agar control and 115 Pa conditions had secondary peaks (Table 1). However, neither was experimentally relevant as both were AR < 2 (i.e. not elongated). These data further illustrate GBM cell response to CSS: a dramatic response associated with an induced elongated cell subpopulation in LN229 cells and a minimal response independent of cell elongation below 115 Pa in U251 cells.

**Table 1:** Lognormal Curve Fitting Parameters of Aspect Ratio (AR) Distributions.

		1° Peak Mode	2° Peak Mode	R^2^
LN229	Control	1.29	2.12	0.94
	Agar Control	1.32	2.19	0.98
	23 Pa	1.37	2.63	0.91
	47 Pa	1.24	2.66	0.99
	115 Pa	1.19	—	0.95
U251	Control	1.15	—	0.97
	Agar Control	1.18	1.68	0.97
	23 Pa	1.24	—	0.97
	47 Pa	1.14	—	0.98
	115 Pa	1.21	1.63	0.96

### Differential gene expression, correlated to increased migration, is induced at low CSS in LN229 cells

The mechanism by which physiologically relevant levels of CSS transduce increased migration and elongation in GBM cells is presently unclear. To gain a comprehensive perspective of changes induced by CSS, RNA regulation of LN229 cells at 23 Pa in the wound healing assay was compared to that of control cells after 18 hr using the GeneChip™ Human Transcriptome Array 2.0 microarray. Of the 135750 RNAs analyzed, there were 2727 differentially expressed RNAs at 23 Pa compared to the control (p < 0.05, false discovery rate (FDR) p < 0.05, absolute fold change >2) (Supplementary Fig. 3). The three most upregulated RNA were *STC1, TMEM45A*, and *CA9* with fold changes of 12.79, 9.64, and 7.75, respectively (Table 2). These and other differentially regulated RNAs of note (Table 2) are associated with poor prognosis, chemoresistance, recurrence, increased motility, and angiogenesis. Additionally, 27 precursor miRNA were differentially regulated, 11 of which correlate with prognosis, treatment resistance, angiogenesis, proliferation, or invasion in human GBM (Table 3). Implication of *CA9*, *ITGA3*, and *let-7i* may explain increased migration and elongation results observed here^22,23^. However, differential expression of selected RNAs and miRNAs alone does not provide enough context to identify interactions or correlation to clinically relevant outcomes.

**Table 2:** Selected mRNA Differentially Regulated Under 23 Pa CSS.

	Fold change	FDR p-value	Cancer-Related Effects
*STC1*	12.79	0.0002	Poor prognosis^55^
*TMEM45A*	9.64	0.0002	Progression, chemoresistance^56^
*CA9*	7.75	0.0011	Increased motility^57^
*ITGA3*	2.34	0.0245	Motility^58^
*FOXN2*	-2.63	0.0386	Poor prognosis^59^
*Ly6k*	4.83	0.0072	Recurrence^60^, tumor grade^61^
*CTGF*	2.17	0.0314	Angiogenesis, poor prognosis^62^
*VEGFA*	3.02	0.0072	Angiogenesis^63^
*VEGFB*	2.04	0.0177	Angiogenesis^64^

**Table 3:** All miRNA Differentially Regulated at 23 Pa CSS.

miRNA	Fold change	Glioma-Related Effects	miRNA	Fold change
*mir-31-HG*	−2.03	Survival^65^	*mir-563*	−2.38
			*mir-569*	−2.55
*mir-100-HG*	−2.63	Radioresistance^66^	*mir-604*	−3.06
mir-181-A1-HG*	−4.2	Prognosis, Radioresistance ^67,68^	*mir-4477-B*	−2.21
mir-181-B1*	−2.9	Prognosis, Radioresistance ^67,68^	*mir-6125*	−2.2
*mir-421*	−2.05	Invasion, Radioresistance^69^	*mir-6839*	−2.43
*mir-423*	−3.28	Angiogenesis, Chemoresistance^70^	*mir-7978*	−2.78
*mir-454*	−2.22	Prognosis^71^	*mir-8063*	−2.7
mir-548-AJ2*	−2.16	Prognosis, survival ^32,33^	*mir-924*	−3.32
mir-548-AZ*	−2.03	Prognosis, survival ^32,33^	*mir-943*	2.02
mir-548-T*	−2.3	Prognosis, survival ^32,33^	*mir-3614*	2.02
*let-7i*	−3.35	Invasion, Proliferation ^22,23^	*mir-8085*	2.06
			*mir-4640*	2.12
			*mir-6884*	2.19
			*mir-3611*	4.17
			*mir-6787*	4.95

*denotes role of miR family where unique isoform data is unavailable. The miRNA on the left have been previously implicated in GBM and are listed with the known effects. The miRNA on the right are unsubstantiated in the GBM literature.

### Pathway analysis predicts changes in cell function and HIF-1 pathway activation at low CSS

**Figure 3:**
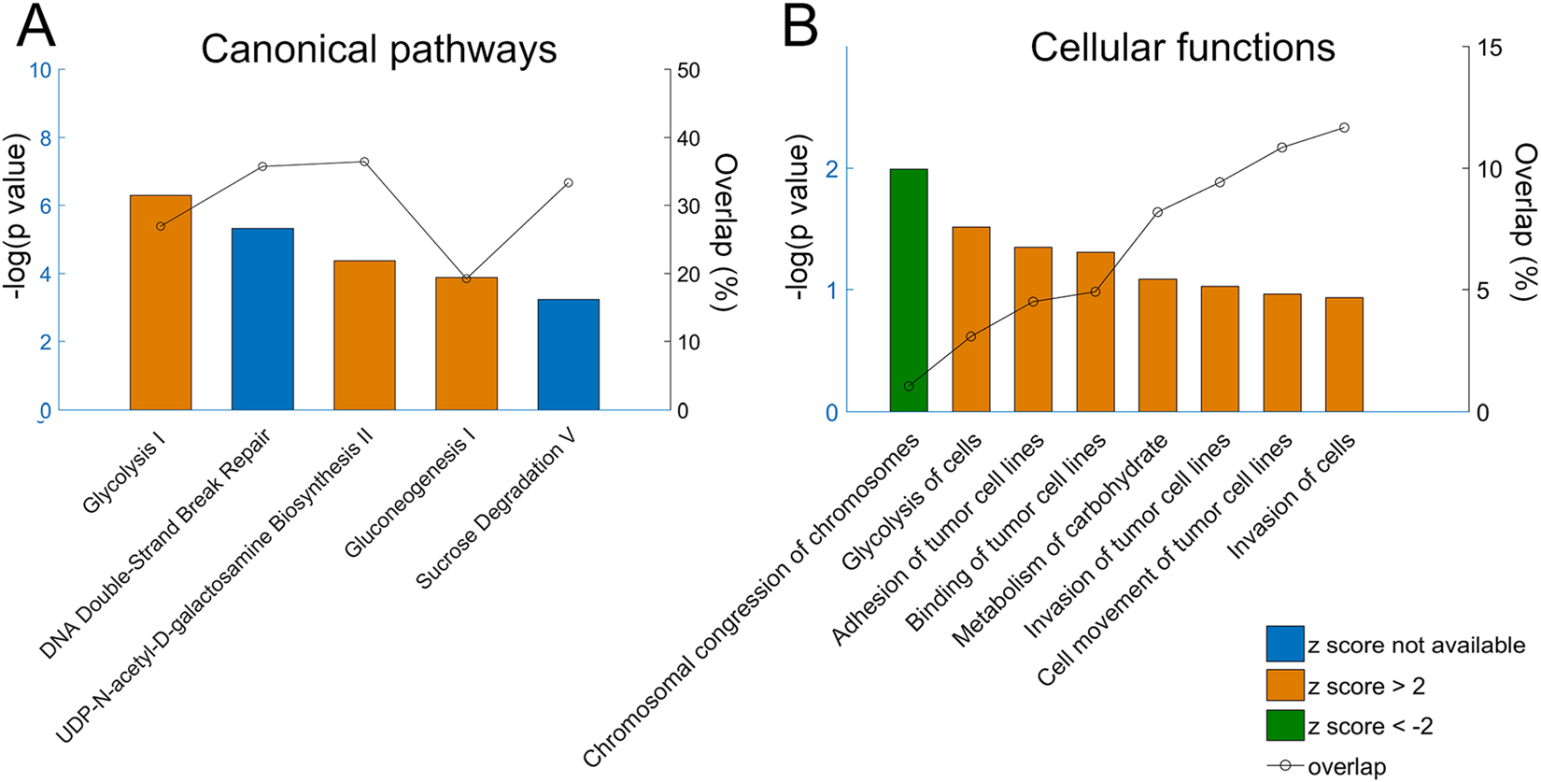
IPA analysis of canonical pathways and cellular functions at low CSS (23 Pa). (**A**) Top canonical pathways ranked by -log(p value). Overlap, indicated on the right axis, is calculated as the number of differentially regulated genes in the pathway over the number of total genes in that pathway. (**B**) Top molecular and cellular functions ranked by -log(p value). Overlap, shown on the right axis, is the number of differentially regulated genes related to that function over total number of differentially expressed genes. Z scores higher than 2 indicate pathway activation/ cell function increase, z scores lower than -2 indicate pathway suppression/ cell function decrease.

To visualize pathways dependent on differentially regulated targets, a total of 489 protein (FDR P < 0.05, FC >2) coding genes were analyzed using Ingenuity® Pathway Analysis (IPA). The analysis compared the differentially regulated gene targets with existing pathway networks and generated 3 major outputs: top canonical pathways, top molecular and cellular functions, and top upstream regulators. There were 45 canonical pathways (P < 0.05, ranked by overlap of target molecules out of total molecules in the pathway) identified to be altered by low CSS at 23 Pa. Within the top canonical pathways, IPA predicted an activated state for gluconeogenesis I (z = 2.236), glycolysis I (z = 2.646), and UDP-N-acetyl-D-galactosamine biosynthesis II (z = 2) as indicated by a z score higher than 2 (Fig. 3). Additionally, IPA predicted cellular movement, cell-to-cell signaling and interaction, and carbohydrate metabolism to be increased (z > 2). Interestingly, chromosomal congression of chromosomes, which is the first step in mitosis, was predicted to be decreased (z = −2), suggesting possible decreases in proliferation (Fig. 3, Supplementary Table 2).

IPA software also predicted upstream regulators activated or inhibited by treatment with 23 Pa CSS. *HIFA* (z = 4.85, p-value = 1.77E-17) and *ARNT* (z = 3.67, p-value = 2.69E-14), which encode the two subunits of hypoxia-inducible factor (HIF)-1, HIF-1α and HIF-1β, respectively, were the top two activated upstream regulators. Additionally, *EGLN* (z = −4.02, p-value = 4.96E-12), a HIF prolyl hydroxylase, was inhibited. Together, this predictions suggests that 23 Pa CSS significantly activates the HIF-1 signaling pathway. HIF-1 is commonly overexpressed in human cancers and is associated with cell apoptosis, proliferation, invasion, and angiogenesis^24,25^. *NUPR1* (z = 4.356, p-value = 1.49E-12) was also predicted to be activated. *NUPR1* is upregulated in response to stress and has a promoting effect on cancer cell metastasis and resistance to therapy^26,27^. These results hint toward a possible mechanism of cell response to low CSS values.

### miRNA-RNA interaction analysis indicate miR548 family in CSS-mediated responses

**Figure 4:**
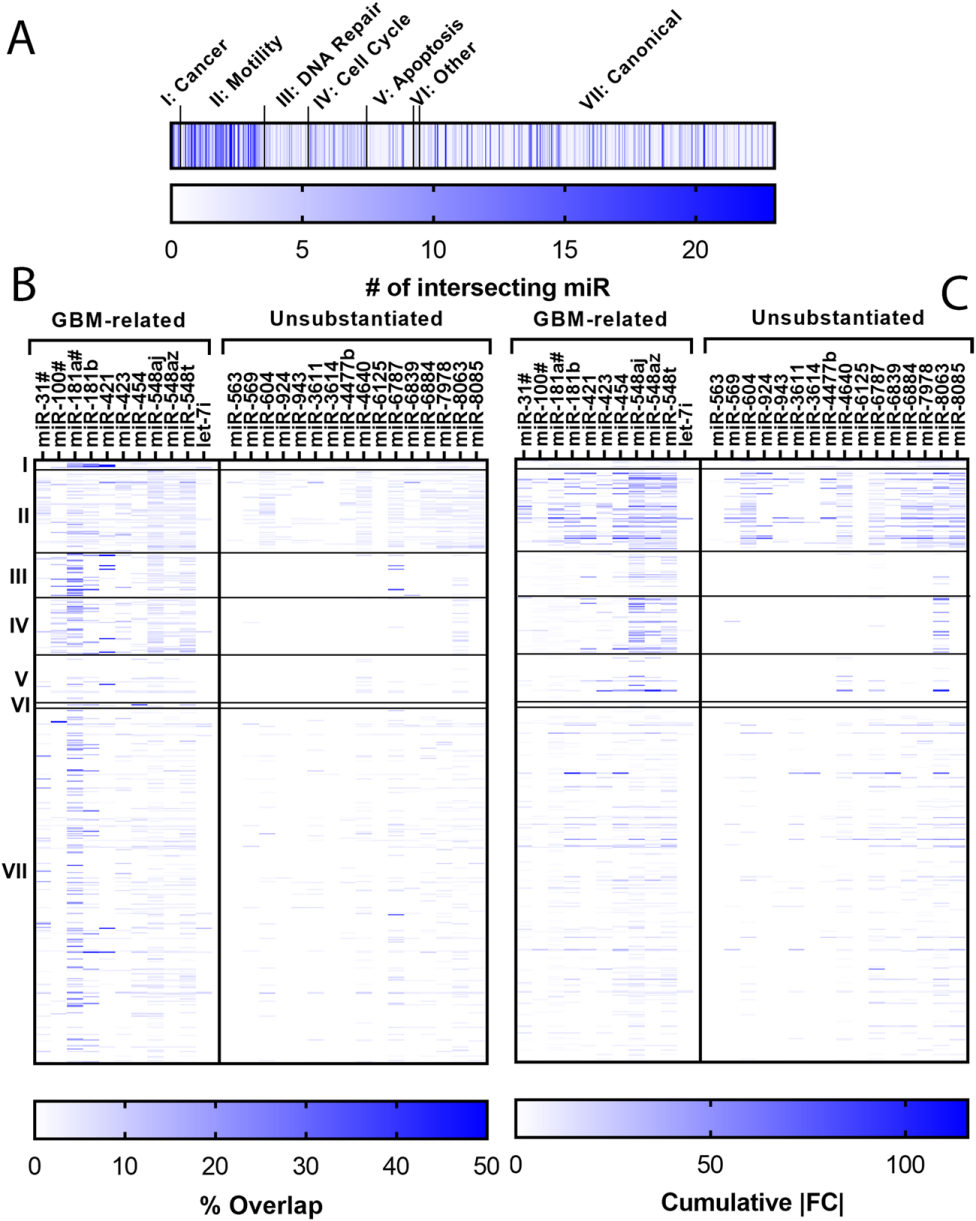
miRNA-mRNA interactions show miRNA and pathway group dysregulation. (**A**) The total number of miRNA with target mRNA that interact with a given pathway. (**B**) Percent overlap for each miRNA-pathway pair. Percent overlap: the % of mRNA targets that were differentially regulated. (**C**) Cumulative absolute fold change (FC) for each miRNA-pathway pair. Cumulative absolute FC: the cumulative unsigned fold change of the differentially regulated targets. A total of 167 pathways were classified into functional groups: I: Cancer, II: Motility, III: DNA Repair, IV: Cell Cycle, V: Apoptosis, VI: Miscellaneous, VII: Canonical. The 27 miRNA were split by their relevance to GBM. miRNA denoted with a hashtag (#) denotes a substitution for a miRNA included in a host gene.

Studies demonstrating the effect of a specific mRNA or miRNA on a cancer-related metric (e.g., invasion) are often performed using viral transfection to demonstrate a correlation. Whereas this is useful for understanding a single gene, it is unclear what, if any, effect will result in the context of a complex microenvironment, such as the changes induced by a mechanical stimulus here. To identify interactions between RNAs and miRNAs identified in microarray analysis and cancer-related metrics, we performed miRNA-RNA interaction analysis. Specifically, we cross-referenced the 27 precursor miRNAs (Table 3) and their differentially regulated target mRNAs identified by microarray analysis with 517 pathways, across 4 databases, representing 7 cancer progression-related classifications, using custom Python scripts (Supplementary material) and miRPathDB^28^. Pathways were classified into one of seven pathway groups using inclusion/exclusion criteria to facilitate analysis (Supplementary Table 3, 4).This analysis yielded various heat maps, with nodes depicting either the number of intersections, percent overlap, or cumulative absolute fold change (FC) (Fig. 4). A miRNA was defined as having an intersection with a pathway if it had significantly more target mRNA in the pathway than expected by random chance (p < 0.05). Percent overlap was defined as the number of differentially regulated target mRNAs normalized to the total number of target mRNAs for a given miRNA-pathway pair. Cumulative absolute FC was the sum of the absolute FC of all differentially regulated target mRNAs for a given miRNA.

The Motility pathway group had the highest number of intersections, with 4 pathways intersecting more than 20 of the 27 miRNA (Fig. 4A). The 4 pathways were cell projection morphogenesis, cell projection, plasma membrane bounded cell projection, and cell projection organization. These are all related to cell projections and may help explain increased elongation seen experimentally. The DNA Repair, Apoptosis, and Miscellaneous pathway groups were not strongly targeted. Increased DNA repair and apoptosis avoidance are linked to treatment resistance, as demonstrated by the effect of MGMT methylation on patient prognosis^29^. *MGMT* encodes a DNA-repair protein that allows cell survival after treatment with alkylating agents (e.g., TMZ)^30^. Microarray results implicated dysregulation of several genes that could be associated with development of resistance to therapy (Tables 2, 3). However, miRNA-mRNA interactions analysis did not implicate DNA repair or apoptosis-avoidance mechanisms, at least over the time points evaluated in this study. Whereas several of these processes can be rapid in individual cells (e.g., minutes to hours), the methods employed here are not sensitive to population-level genetic shifts that may take longer to manifest.

**Figure 5:**
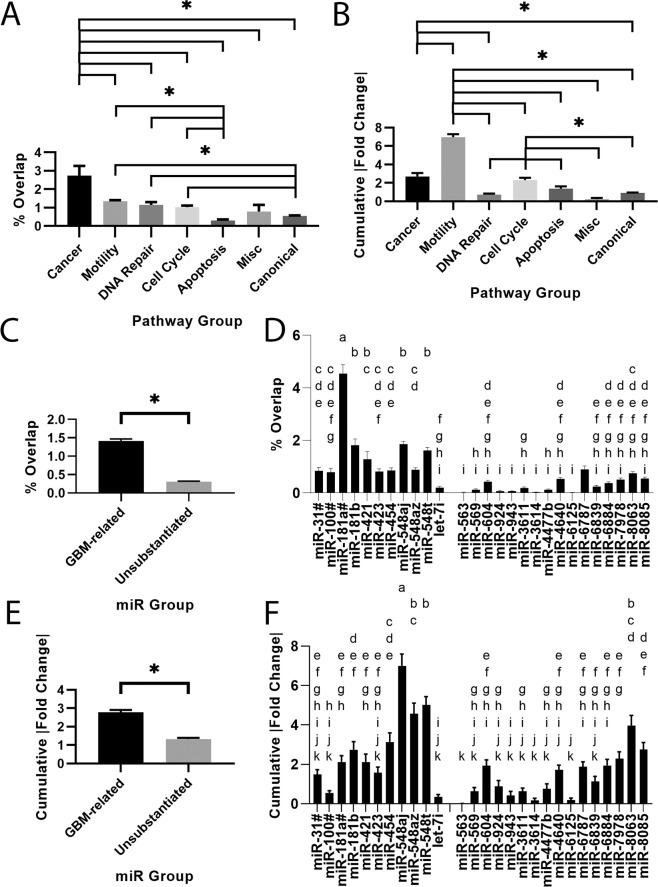
Canonical, Motility, and Cell Cycle Pathway groups and the miR548 family are predicted to be altered under 23 Pa CSS based on mRNA-miRNA interaction analysis. (**A**) Percent overlap and (**B**) cumulative absolute fold change (FC) for each pathway group. (**C**) Percent overlap and (**D**) cumulative absolute FC for each miR group based on literature relevance to GBM. Levels connected by a star (*) are statistically significant at α = 0.05. (**E**) Percent overlap and (**F**) cumulative absolute FC for each miRNA across all pathways. Letters not connected by the same letter are statistically different at α = 0.05. miRNA denoted with a hashtag (#) denotes a substitution for a miRNA included in a host gene.

Percent overlap (Fig. 4B) and cumulative absolute FC (Fig. 4C) were used to quantify the influence each miR exerted over a given pathway in terms of quantity and quality, respectively. The Cancer pathway group was used as a standard for comparison, as these pathways are expected to be strongly dysregulated in GBM. Cancer had the highest percent overlap (Fig. 5A), as expected, but was eclipsed by the Motility pathway group with respect to cumulative absolute FC (Fig. 5B). Percent overlap for the Motility pathway group was about half as large as the Cancer pathway group, whereas cumulative absolute FC was ~2.5x larger. Significant dysregulation of the Motility pathway group was expected based on migration and morphology results. These results suggest that increased motility observed under CSS is related to strong differential regulation of fewer genes. The Cell Cycle pathway group also demonstrated relatively high dysregulation compared to the Cancer pathway group. Percent overlap for the Cell Cycle pathway group was around half as large as the Cancer pathway group, similar to the Motility pathway group. However, its cumulative absolute FC was similar to the Cancer pathway group (2.32, 2.69, respectively). This result corroborates IPA results, suggesting altered proliferation at 23 Pa CSS. Additionally, miRNAs were classified based on their relevance to GBM in peer-reviewed literature, indicating a significantly strong influence as measured by percent overlap and cumulative absolute FC (Fig. 5C,E). This result hazards caution when extending these data to other cancers, at least in the context of mechanobiology.

The maximum percent overlap was 50% for the WikiPathways ATM Signaling Network in Development and Disease. The maximum cumulative absolute FC was 115.98 in the GO Biological Process Apoptotic Process. Although these were maxima are in the Canonical and Apoptosis pathway groups, respectively, neither pathway group as a whole was strongly affected. In grouped analysis, the Canonical and Apoptosis pathway groups demonstrated the lowest percent overlap, less than ~0.55 compared to the Cancer pathway group at 2.74. Their cumulative absolute FC was also less than roughly half of the Cancer pathway group at 2.69. These results suggest that altered apoptosis is not relevant in this model at this time point.

The influence of each miRNA was also quantified as a means of identifying potential therapeutic targets. By percent overlap, mir-181a had the strongest influence, followed by *mir-548aj, mir-181b*, and *mir-548t* (Fig. 5D). By cumulative absolute FC, *mir-548aj, mir-548t*, and *mir-548az* had the strongest influence (Fig. 5F). Downregulation of the *miR181* family is associated with glioma progression^31^, whereas the *miR548* family is associated with prognosis and survival^32,33^. These results suggest that both families are potential therapeutic targets. However, *mir-181A1HG* was detected in the microarray and *mir-181a* was substituted to match isoform data availability (Fig. 4). This warrants caution, as host gene regulation may not necessarily match that of the miRNA^34^. These results suggest that the miR548 family exerts a strong influence on pathway groups implicated in GBM response to CSS and may be the best candidate for pharmaceutical intervention.

## Discussion

This study presents initial work toward identification of pathways involved in GBM response to CSS, particularly at levels (i.e., <115 Pa, or roughly half of maximum observed in a mouse model^18^) that might be observed in the 2 cm region of recurrence adjacent to the tumor periphery. This study also examined the relationship between tumor-induced physical force and aggressive tumor features to identify possible positive feedback loops. Specifically, cell migration, morphology, and differential gene signaling were examined for well-characterized LN229 and U251 GBM cell lines. Both LN229 and U251 cell lines exhibited maximal migration at applied CSS of 23 Pa, compared to little/no statistical difference observed for lower pressures indicative of a “normal” brain environment (Fig. 1). According to the best available approximation of CSS distribution through the brain^12^, GBM cells experience this level of CSS at 5-10% of the tumor radius away from the tumor. As such, it is possible that CSS at the tumor periphery temporarily “boosts” GBM cell migration. These results are consistent with previous findings. GBM cells display high sensitivity to their mechanical microenvironment. For example, we have shown that U87 GBM cells grown on 50 µm thick electrospun fiber mats^35^ or patient-derived GBM cells cultured in <25 µm thick hydrogels^36^ are sensitive to the mechanics of their underlying supports. GBM cells also increase migration in response to low linear flow rates (<1 µm/s)^37^.

Increased migration at 23 Pa likely results from cross-talk between mechanically-responsive integrins and metabolic pathways. Microtubules resist and dissipate the CSS mechanical load, transmitting force to the extracellular environment via focal adhesions^38^. At focal adhesions, mechanical forces converge with biochemical signaling, initiating signal transduction^38^. There is significant cross-regulation between integrins and metabolic pathways (i.e., *AMPK, mTOR, HIF-1*)^39^. In particular, integrins contribute to and are necessary for differential signaling in cancer cells that leads to increased glycolysis and biosynthetic precursor production^39,40^. We detected a significant upregulation of *ITGA3* (FC = 2.34, FDR P = 0.0245), encoding integrin α_3_, in cells treated with CSS. Upregulation of integrin α_3_ has been detected in cells treated with elevated CSS^14^ and hydrostatic pressure^41^. The downstream signaling of integrin α_3_ is illustrated in the focal adhesion network, as generated from WikiPathways (Supplementary Fig. 4).

We also observed differences in migratory behaviors between the two cell lines. For LN229 cells, wound closure decreased below that of the control at high levels of CSS, whereas for U251 cells wound closure was equivalent to or higher than the control at all CSS values investigated. These differences in migratory response were also correlated to morphology. We observed a mechanically-induced, elongated subtype in LN229 cells, present at all pressures except the highest employed, but that was most pronounced at 23 Pa CSS (Fig. 2). This subtype was not observed in U251 cells (Table 1). This behavior may be similar to the collective migration leader cell phenotype observed in breast cancer cell compression studies, which is induced by forced filopodia extrusion into the cell-free area on one side of the cell^13^. However, this is unlikely. Our study employed non-confluent, monolayer culture in which cell-free area was available on all sides. Additionally, we neither observed nor measured leader cell formation in wound healing assays. Previous studies also used much higher levels of CSS (i.e., 773 Pa vs 0-115 Pa here), which was physiologically relevant to much stiffer breast cancer tissue. As such, the degree of forced extrusion into the cell-free area would be proportionally lower. The elongated morphologies observed here may be adopted simply as a result of their energetic favorability; an elongated cell has a lower projected cell area, minimizing CSS versus a round cell. However, LN229 cells did not uniformly elongate, instead forming a secondary peak with specific AR maximum of ~2.6 (Table 1). This behavior suggests a phenotypic change. More experiments are needed to understand differences in single cell and collective cell responses to CSS in LN229 cells. Further, it is important to note that this study examined isolated GBM cell response, whereas the tumor microenvironment is a complex niche with many interconnected cues. Thus, the collective tumor response may differ from that of isolated GBM cells and can best be discerned by increasing model complexity with the addition of stromal or immune cells or through *in vivo* studies. Such studies would elucidate differences between isolated GBM response and effects of cell-cell signaling. *In vivo* studies would be particularly informative as CSS is likely distributed through many structural elements aside from GBM cells, such as blood vessels, extracellular matrix, and stromal cells.

Physiologically, GBM cells elongate by sending out long projections along white matter tracts and the perivascular space^42^. Thus, the observed correlation of elongation and increases in migration for LN229 cells (Figs. 1, 2) may result from activation of shared pathways. To identify potential pathways involved in GBM CSS response, we assessed LN229 cell signaling at 23 Pa versus controls with no applied CSS using a Human Transcriptome Array. Changes in several mRNA and precursor miRNA associated with tumor aggressiveness were observed (Tables 2, 3). Low CSS level at 23 Pa was predicted to upregulate cell carbohydrate metabolism through glycolysis by IPA analysis (Fig. 3). The gluconeogenesis pathway regulates production of glucose, and the glycolysis pathway leads to the breakdown of glucose for energy generation. Known as the Warburg effect, cancer cells favor glycolysis, which produces less ATP than oxidative phosphorylation, in the metabolism of glucose^43^. Glycolysis in GBM is upregulated (~3 times higher than the normal brain)^44^ and promotes cell survival by suppressing apoptosis^45^. Glycolysis may be even more favored under hypoxia, as shown in GBM stem-like cells^46^. The upregulation of glycolysis suggests enhanced energy generation in GBM cells treated with CSS at 23 Pa.

IPA analysis also identified *HIFA* and *ARNT*, encoding the two subunits HIF-1α and HIF-1β of HIF-1, as the top upstream regulators activated by low level of CSS. HIF-1 is commonly overexpressed in human cancers and is associated with cell apoptosis, proliferation, invasion, and angiogenesis^24,25^. Although the production of HIF-1 is most commonly induced by hypoxia, growth factors and hormones can also induce HIF-1 in a normal oxygen environment^47^. Moreover, emerging evidence suggests that mechanical forces can trigger HIF-1 induction. For example, HIF-1α can be induced by mechanical stretching in capillary endothelial cells^48^, by compression in skin cells^49^, and by pressure in heart ventricles^50^. HIF-1α is also upregulated in the brain cortex following traumatic brain injury^51^. Our results suggest that HIF-1 most likely plays a role in cell response to CSS.

To provide context with known signaling pathways, we cross-referenced differentially regulated miRNAs with known targets and multiple databases to validate and predict phenotypic behaviors (Figs. 4, 5). Use of multiple databases and hand-tagged pathway groups (Supplementary Table 3) enabled predictions and correlations independent of a single database or pathway. For each pathway, we analyzed percent overlap, which indicates the percent of target mRNAs that were differentially regulated in a given pathway, as well as the cumulative absolute FC, which is the summed magnitude of signaling change for all mRNAs in a given pathway. Percent overlap is a normalized measured of how many target mRNA in a given pathway were differentially regulated. Conversely, cumulative absolute FC is a measure of how significantly each pathway was differentially regulated independent of numbers of target mRNAs effected.

Unsurprisingly, the Cancer pathway group, containing classical pathways related to cell signaling in cancer, was the most influenced pathway group by percent overlap (Figs. 4B, 5A) (i.e., numbers of mRNA). We used the Cancer pathway group as a relative marker to understand the significance of changes in other pathways relevant to GBMs. For example, the KEGG Glioma pathway has several signaling outcomes, including migration, proliferation, and survival. The inclusion of the additional pathway groups helps predict which outcomes are likely to occur in this model. The Motility and Cell Cycle Pathway groups were the most strongly influenced (Fig. 5A,B). Given their low %overlap, this suggests that, for these pathways, downstream transduction is implemented by larger changes in expression of a few RNAs as opposed to small changes in many RNAs. Both of these pathways were also highlighted by IPA (Fig. 3), validating experimental observations and providing strong prediction of cell cycle regulation changes in response to CSS. IPA also suggested a decrease in proliferation pathways (Fig. 3). GBM cells are typically dichotomous, exhibiting either migratory or proliferatory phenotypes^52^. Apoptosis is often suppressed in migrating GBM cells^53,54^, making them less susceptible to anti-proliferative therapies. However, we observed increased migration with predicted decreases in proliferation without significant changes in the Apoptosis pathway group (Fig. 5A,B). This may simply be a result of the short duration of this study (i.e., 18 hr exposure to CSS) or may indicate more complex signaling. Increased migration into the brain parenchyma enables cells to escape focal treatments (i.e., surgical resection, radiotherapy), whereas decreased proliferation correlates with suppressed apoptosis. Thus, these findings are consistent with features of tumor aggressiveness. The heterogeneous cellular response to CSS, with LN229 displaying increased migration at low CSS and decreases at high CSS, whereas U251 cells were relatively insensitive to compression, indicates potential for both patient-to-patient variability in CSS response as well as variability within individual tumors. This variation likely depends on the genetic signature of each cell type, but indicates the potential for formation of an aggressive, migratory phenotype with poor therapeutic response to GBM drugs primarily targeting proliferating cells.

Interaction analysis identified some miRNA signaling molecules important in the CSS response. The *miR548* family consisting of *mir-548aj, mir-548az*, and *mir-548t* demonstrated the largest influence, despite relatively low FC (i.e., −2.16, −2.03, and −2.3, respectively) (Fig. 5E,F). These FCs were in the bottom half of absolute FC among all 27 differentially regulated precursor miRNA (Table 3). The high cumulative absolute FC of the mir-548 family suggests that regulation likely occurs via large changes in a small number of target mRNA. Compared to normal brain tissue, the *miR548* family is typically downregulated in GBM, suggesting a protective, prognostic role in the healthy brain^32^. Expression also correlates with survival, with mir548d-3p/-5p upregulated in long-term survival patients^33^. This correlation is a critical finding as, to the best of our knowledge, there are currently no recognized miRNAs linked to GBM mechanobiology. Thus, these data provide an important first step toward mining the mechanical tumor microenvironment for potential drug targets. Further studies in models of increased microenvironment complexity (e.g., stromal, immune cells, extracellular matrix components, 3D culture), that investigate downstream interactors, and genetic confirmation of miR548 activity would strengthen these claims. With experimental evidence provided in this mechanobiology study and in gross tumor studies by others^32,33^, the miR548 family may be a candidate for pharmaceutical intervention when targeting this microenvironment niche. These data thus provide a candidate group for pharmaceutical intervention (i.e., the *miR548* family) identified by leveraging the transcriptome, a cornucopia of signaling literature, and miRNA-mRNA interactions.

## Materials and Methods

### Cell culture

Two commonly used glioblastoma cell lines, LN229 and U251 (ATCC), were employed to study the effects of compression in GBM and provide concordance with our previous results. These cell lines were cultured using standard procedures. Cells were fed 2-3 times per week with DMEM/F12 media (Sigma-Aldrich, St. Louis, MO, USA) supplemented with 10% fetal bovine serum (Fisher Scientific, Hampton, NH, USA), 1% penicillin/streptomycin (Invitrogen, Carlsbad, CA, USA) and 1% MycoZap (Life Technologies, Carlsbad, CA, USA).Cells were passaged at ~80% confluency.

### Mechanical compression model

CSS was created using a modified version of the model described in Tse *et al*.^13^ However, unlike that study in which cells were cultured in a semi-confluent and patterned monolayer, cells in our model were cultured in a non-confluent monolayer. Cells were therefore compressed in 1D, similar to radial forces experienced by GBM cells. Briefly, cells were cultured on the membrane of a Transwell© insert (VWR® tissue culture plate inserts, polyester membrane, 12 mm diameter, 0.4 µm pore size) in a 12-well tissue culture plate (VWR). Cell culture media was added to the inner well. This pore size was selected to allow nutrient and oxygen exchange and prevent transmigration across the membrane. An agar cushion was placed atop the cells to protect against edge effects^36^ and from contact with the rigid weight discs used to create CSS. To prepare the agar cushion, 1 wt% agar solution was heated at 90 °C for ~5 min or until translucent. This solution was then poured into a 100 mm cell culture dish, allowed to set for ~5-10 min, and sterilized under ultra-violet (UV) light for 20 minutes. The secured agar gel was punched out to a size ~ 3.5 mm thick and 10 mm in diameter with an arch punch (Grainger, Columbus, OH, USA). Finally, varying numbers of weight discs made of stainless steel, aluminum, or vinyl were placed on top of the agar cushion to apply a known compression level (Supplementary Fig. 1A). Specifically, compressions of 13, 23, 47, and 115 Pa were employed, with an error of approximately ± 0.5 Pa. Control conditions included samples with no weight or with agar cushions only. To prevent disc oxidation or other disc-related changes in cell phenotype, discs were coated with Silastic (Dow Corning). Weight discs were sterilized in ethanol under UV for 40 min and dried completely prior to use.

### Wound healing assay

Collective migration was assessed using the wound healing gap assay. An Ibidi® 2 well chamber-insert (Ibidi 81176) was used to pattern a consistent 500 µm-wide cell-free gap on the Transwell© membrane. Cells were stained with CellTracker Green; 2 × 10^5^ cells were seeded in 70 µL full media in each side of the Ibidi chamber-insert and allowed to adhere for 6 hr. Then, 1 mL of media was added to the outer well of the Transwell© insert, and the Ibidi chamber was removed. Agar cushions and weight discs were then added to create the desired mechanical compression. One image per insert was taken at 4x magnification in the same location at 0 hr and 18 hr after culture using an Olympus IX 71 inverted fluorescence microscope with a FITC filter. The cell-free area was quantified at the initial and final time points using ImageJ (Supplementary Fig. 1B,C), and wound closure calculated according to Eq. 1. To account for culture variations, differential wound closure is reported, which is the average difference in wound closure between each compression condition and the control (Eq. 2).

1 $$Wound\,Closure\,\,(WC)=\frac{Are{a}_{initial}-Are{a}_{final}}{Are{a}_{initial}}$$

2 $$\Delta Wound\,Closure=\overline{W{C}_{exp}-W{C}_{ctrl}}$$

### Morphological analysis

Cells were stained with CellTracker Green (CMFDA, Invitrogen), seeded at 1 × 10^5^ cells per Transwell© insert (N = 3 per condition) in a 12 well plate, and allowed to adhere for 24 hr. Then, an agar cushion was applied (for agar control and compression conditions), and weight discs were added (for compression conditions) to obtain the desired compression. After 18 hr of applied CSS, cells were imaged at 20x in 3 random locations per insert using an inverted fluorescence microscope with a FITC filter (Olympus IX 71). Cell morphology was characterized using Image J to determine the aspect ratio (AR) and area of cells in each images. Incomplete cells (on the edge) or dividing cells were excluded.

AR and cell area histograms were plotted in MATLAB to detect distribution patterns. AR distribution was plotted and fit to 1-term or 2-term probability density function (PDF) using the curve fitting tool in MATLAB with the following equations:

3 $${x}={AR}-1$$

4 $$1-{\rm{term}}\,{\rm{PDF}}:f(x)=a1\times \frac{1}{x}\times \frac{1}{\sigma 1\sqrt{2\pi }}\exp (-\frac{{(lnx-\mu 1)}^{2}}{2\sigma {1}^{2}})$$

5 $$2-{\rm{term}}\,{\rm{PDF}}:f(x)=a1\times \frac{1}{x}\times \frac{1}{\sigma 1\sqrt{2\pi }}\exp (-\frac{{(lnx-\mu 1)}^{2}}{2\sigma {1}^{2}})+a2\times \frac{1}{x}\times \frac{1}{\sigma 2\sqrt{2\pi }}\exp (-\frac{{(lnx-\mu 2)}^{2}}{2\sigma {2}^{2}})$$

X in Eq. 1 was set to be AR-1 because the value of AR is at least 1. Scaling parameters, a1 and a2, determine the percentage of each fitted equation. The distribution curve means and standard deviations are given by µ1, µ2 and σ1, σ2, respectively. The mode (m), which represents the peak location of each curve, is determined by:

6 $${\rm{m}}=\exp (\mu -{\sigma }^{2})+1$$

Goodness of fit was compared by the value of sum of squares for error (SSE) and R^2^ reported by MATLAB. The fitting that resulted in the smaller SSE and higher R^2^ was presented.

### RNA extraction

LN229 cells treated with no compression or compression at 23 Pa for 18 hr were processed for microarray analysis. Briefly, LN229 cells were patterned using the Ibidi chambers and cultured in a Transwell© insert without staining as described above. To ensure even compression over the whole cell population, one side of the Ibidi chamber was cut off, placed in the center of the Transwell© membrane, and used for cell patterning. No compression was added to the control. After 18 hr of incubation, Transwell© inserts were washed 3x with PBS and immersed in TRIzol reagent (ThermoFisher 15596026) for RNA extraction following the manufacturer’s instructions. Extracted RNA was further purified with an RNA clean-up and concentration kit (Norgen 23600) and tested for quality by Nanodrop analysis and a TapeStation RNA Bioanalyzer. RNA samples with a 260 nm/230 nm absorbance ratio > 1, a 260 nm/280 nm absorbance ratio > 1.9, and a RNA integrity number (RIN) > 7 were used for microarray analysis as recommended by the manufacturer. Experimental replicates (N = 4 for each group) were prepared in individual inserts and treated with 23 Pa of compression on different days.

### Microarray and pathway analysis

Microarray analysis was performed using the GeneChip™ Human Transcriptome Array 2.0 (Applied Biosystems^TM^) following manufacturer’s instructions. Briefly, 100 ng RNA was used to generate ss-cDNA according to the manufacturer’s protocol in the GeneChip® WT PLUS Reagent Kit, and 15 µg of labeled, fragmented, and biotin-labeled ss-cDNA was hybridized at 45 °C for 16 hr at 60 rpm in an Affymetrix 645 Hybridization oven. GeneChips were then washed and stained using an Affymetrix 450 Fluidics Station, and scanned with an Affymetrix 3000 7 G GeneChip Scanner. Data files were generated and processed with an Affymetrix software and Expression Console. The RNA quality test and microarray were performed by the genomics core at The Ohio State University. Gene expression analysis was performed using the Ebayes ANOVA method in the Transcriptome Analysis Console (TAC 4.01, ThermoFisher) with false discovery rate (FDR) adjusted p < 0.05. Heat maps of gene expression were generated with hierarchical clustering. A total of 489 differential expressed genes (FDR < 0.05, FC > 2) were uploaded into the Ingenuity Pathway Analysis server (http://www.ingenuity.com) to identify pathways/disease/functions regulated by low CSS.

### miR-mRNA interaction analysis

For each precursor miRNA differentially regulated in the microarray, an Excel file was downloaded from miRPathDB that included statistically significant, over-represented pathways and target mRNA in the pathway. If unavailable, miRNA were substituted for a similar sequence or identical sequence. For example, hsa-mir-548-aj was substituted for mir-548-aj2. The ‘2’ in mir-548-aj2 indicates different precursor miRNA that yield identical sequences. Both 5p/3p isomiRs were included to match HTA 2.0 coverage. Then, pathways of interest were hand-tagged into pathway groups classified as Cancer, Motility, DNA Repair, Cell Cycle, Apoptosis, Miscellaneous, or Canonical (Supplementary Table 3) using explicit inclusion/exclusion criteria to avoid bias (Supplementary Table 4). Using Python scripts (Supplementary File 1–8), each miRNA excel file was first cross-referenced with the list of differentially regulated mRNA from the microarray to annotate the file with targets in each pathway that were differentially regulated. Then, the pathway groups were consolidated into a single list with duplicates removed. Finally, heat maps were generated for the number of intersections, percent overlap, and cumulative absolute FC. For number of intersections, each node is the number of intersections for each pathway. For percent overlap and cumulative FC, each node of the corresponding heat map is the intersection of a miRNA and a single pathway. Percent overlap is the number of differentially regulated target mRNA in the pathway normalized to the number of total target mRNA in the pathway. Cumulative absolute FC is the sum of the absolute FC for each differentially regulated target mRNA in the pathway. Individual node values were used for averages for mir groups, pathway groups, and miRNA.

### Statistical analysis

All data were presented as mean with standard error. Wound healing experiments were conducted as matched pair experiments between the experimental condition and the control for each replicate. This allowed us to account for day-to-day variability. Post-hoc one-way t-test α levels were adjusted with Bonferroni corrections to 0.01 for 5 comparisons. The remainder of the data was compared using ANOVA and the Tukey-Kramer HSD method. These analyses were performed with a significance level of α = 0.05. All data was analyzed in JMP Pro 14 statistical software.

## Supplementary information

Supplementary Tables and Figures.
