# Supplementary material for: MicroRNA-mRNA Interactions at Low Levels of Compressive Solid Stress Implicate mir-548 in Increased Glioblastoma Cell Motility
Source: Sci Rep. 2020 Jan 15;10:311. doi: 10.1038/s41598-019-56983-x (PMC6962377; doi:10.1038/s41598-019-56983-x)
Supplement: Supplementary file 1 — Supplementary Tables and Figures. [file 41598_2019_56983_MOESM1_ESM.docx]

Supplementary Information for MicroRNA-mRNA Interactions at Low Levels of Compressive Solid Stress Implicate miR-548 in Increased Glioblastoma Cell Motility

Mark A. Calhoun^1,5^, Yixiao Cui^1,5^, Eileen E. Elliott^2^, Xiaokui Mo^4^, Jose J. Otero^3^, Jessica O. Winter^1,2*^

^1^ Department of Biomedical Engineering, The Ohio State University, Columbus, OH

^2^ William G. Lowrie Department of Chemical and Biomolecular Engineering, The Ohio State University, Columbus, OH

^3^ Department of Pathology, The Ohio State University, Columbus, OH

^4^ Center for Biostatistics and Bioinformatics, The Ohio State University, Columbus, OH

^5^ These authors contributed equally to this work.

*Corresponding author

Correspondence:

Prof. Jessica O. Winter

William G. Lowrie Department of Chemical and Biomolecular Engineering,

Department of Biomedical Engineering,

The Ohio State University,

453 CBEC

151 W. Woodruff Ave., Columbus, OH, 43210, USA

[winter.63@osu.edu](mailto:winter.63@osu.edu)

**Supplementary Figures**


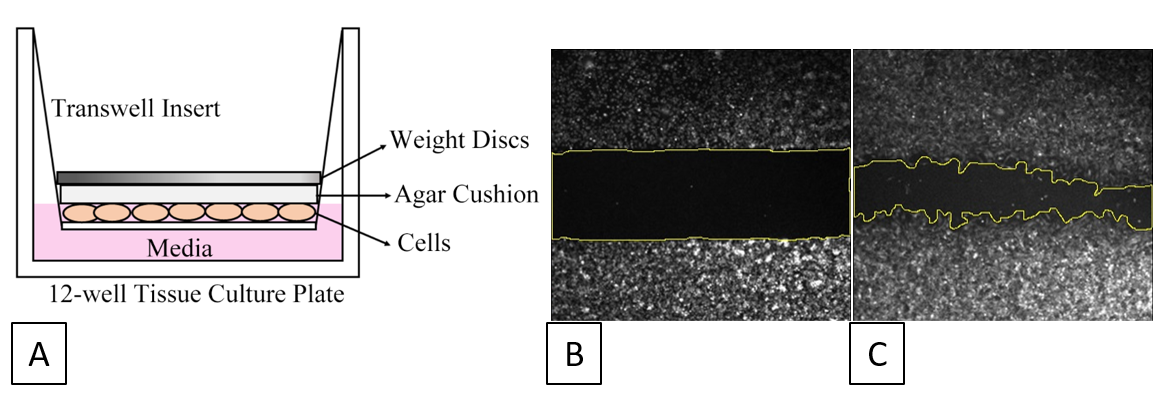


Supplemental Figure 1. Methodology of compressive solid stress experiments. (A) Glioblastoma cells were grown on a Transwell Insert to facilitate access to media and oxygen. Then, an agar cushion and discs of varying weight were placed atop the cells to generate mechanical compression. For wound healing experiments, the initial (B) and final (C) cell areas were used to calculate wound closure percent.


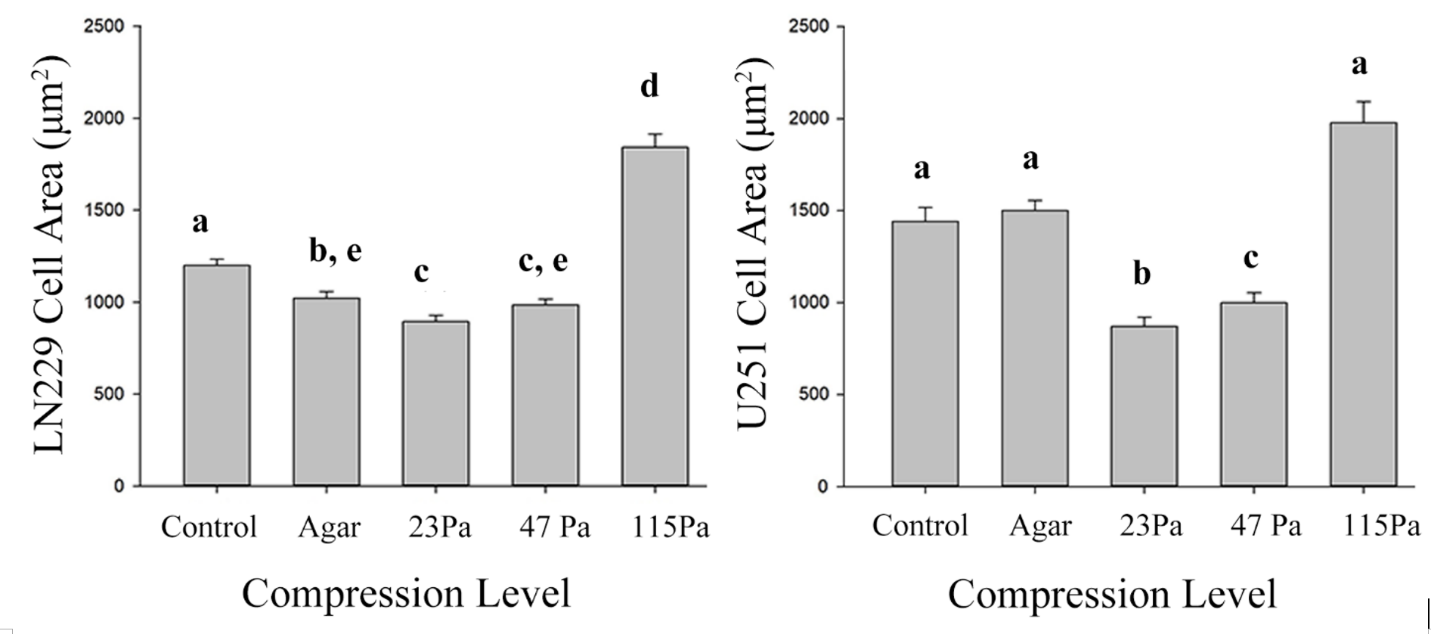


Supplemental Figure 2. CSS decreases cell area at low to intermediate levels (23 and 47 Pa), but increases cell area at high levels (115 Pa). Different letters indicate statistical differences.


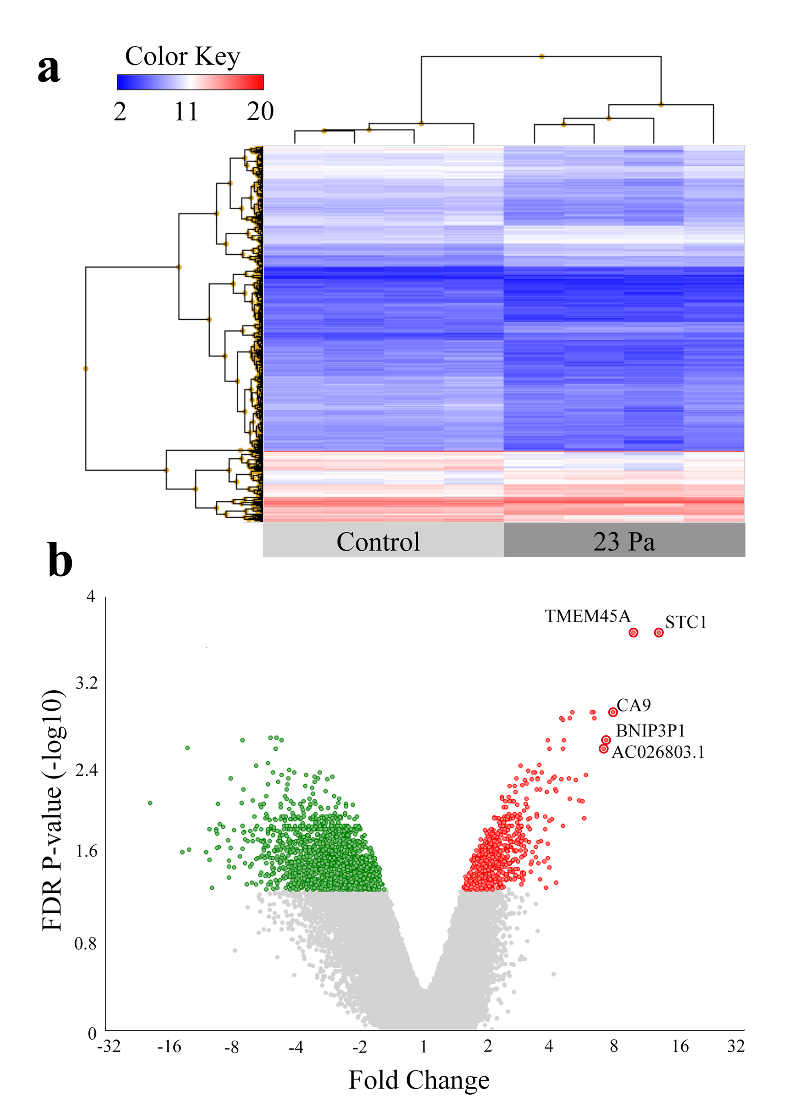


Supplementary Figure 3. Summary of gene expression analyzed by microarray analysis. (**a**) Hierarchical clustering of 2727 differentially expressed genes (P < 0.05, FDR P < 0.05) in control cells and cells treated with 23 Pa CSS. Color key indicates the normalized expression value of each gene. (**b**) Volcano plot of downregulated (green) and upregulated (red) genes in cells at 23 Pa compared to the control. The top 5 upregulated genes are labeled on the figure. Only genes with FDR P < 0.05 are shown in color.

**
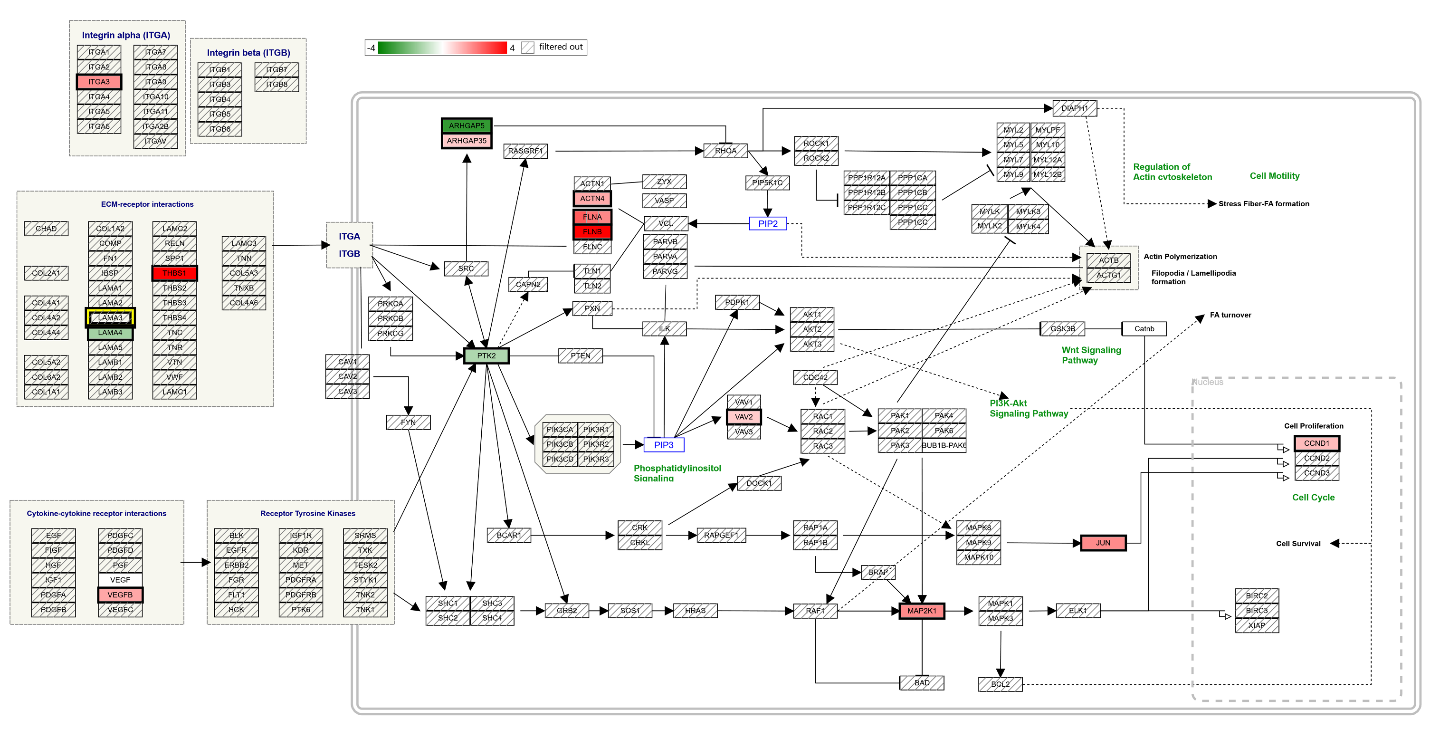
**

Supplementary Figure 4. Gene network of focal adhesion generated by Wikipathways showing upregulated (red) and downregulated (green) genes at CSS of 23 Pa.

**Supplementary Tables**

Supplementary Table 1. Lognormal Curve Fitting of Aspect Ratio (AR) Distributions

|  |  | Primary Peak | | | | Secondary Peak | | | |  |
| --- | --- | --- | --- | --- | --- | --- | --- | --- | --- | --- |
|  |  | a1 | µ1 | σ1^2^ |  | a2 | µ2 | σ1^2^ |  | **R^2^** |
| LN229 | Control | 47.67 | -0.51 | 0.74 |  | 1.10 | 0.12 | 0.0016 |  | 0.94 |
|  | Agar | 39.58 | -0.26 | 0.86 |  | 3.57 | 0.18 | 0.0121 |  | 0.98 |
|  | 23 Pa | 47.02 | -0.17 | 0.83 |  | -1.06 | 0.49 | 0.0001 |  | 0.91 |
|  | 47 Pa | 56.91 | -0.73 | 0.67 |  | 1.34 | 0.51 | 0.0016 |  | 0.99 |
|  | 115 Pa | 8.77 | -1.28 | 0.40 |  | - | - | - |  | 0.95 |
| U251 | Con | 26.39 | -1.13 | 0.79 |  | - | - | - |  | 0.97 |
|  | Agar | 27.21 | -1.37 | 0.32 |  | 27.91 | -0.03 | 0.3481 |  | 0.97 |
|  | 23 Pa | 62.52 | -0.52 | 0.92 |  | - | - | - |  | 0.97 |
|  | 47 Pa | 53.94 | -0.66 | 1.32 |  | - | - | - |  | 0.98 |
|  | 115 Pa | 8.31 | -1.32 | 0.22 |  | 1.85 | -0.45 | 0.0049 |  | 0.96 |

a1, a2= mode; µ1, µ2 = mean; σ1^2^, σ2^2^ = variance, and R^2^=correlation coefficient

Supplementary Table 2. Predicted activation state of cell functions at low CSS (23 Pa)

| Categories | Functions | p-Value | Predicted Activation State | # Molecules |
| --- | --- | --- | --- | --- |
| Cellular Movement | Cell movement of tumor cell lines | 0.000015 | Increased | 53 |
|  | Invasion of cells | 2.98E-07 | Increased | 57 |
|  | Invasion of tumor cell lines | 2.6E-06 | Increased | 46 |
| Cell-To-Cell Signaling and Interaction | Adhesion of tumor cell lines | 2.89E-05 | Increased | 22 |
|  | Binding of tumor cell lines | 0.000234 | Increased | 24 |
| Carbohydrate Metabolism | Glycolysis of cells | 3.19E-07 | Increased | 15 |
|  | Metabolism of carbohydrate | 1.47E-05 | Increased | 40 |
| Cellular Assembly and Organization | Chromosomal congression of chromosomes | 3.16E-06 | Decreased | 5 |

Supplementary Table 3. Pathway Groups

| Database | Pathway | Cancer | Motility | DNA Damage | Cell Cycle | Apoptosis | Miscellaneous | Canonical |
| --- | --- | --- | --- | --- | --- | --- | --- | --- |
| WikiPathways | Senescence and Autophagy in Cancer | • |  |  |  |  |  |  |
| KEGG | Pathways in cancer | • |  |  |  |  |  |  |
| KEGG | Proteoglycans in cancer | • |  |  |  |  |  |  |
| KEGG | Glioma | • |  |  |  |  |  |  |
| WikiPathways | Integrated Cancer Pathway | • |  |  |  |  |  |  |
| WikiPathways | Signaling Pathways in Glioblastoma | • |  |  |  |  |  |  |
| KEGG | MicroRNAs in cancer | • |  |  |  |  |  |  |
| GO Bio Pro | microtubule anchoring |  | • |  |  |  |  |  |
| GO Bio Pro | filopodium assembly |  | • |  |  |  |  |  |
| GO Bio Pro | regulation of actin filament bundle assembly |  | • |  |  |  |  |  |
| GO Bio Pro | cell migration |  | • |  |  |  |  |  |
| GO Bio Pro | regulation of cell projection assembly |  | • |  |  |  |  |  |
| GO Bio Pro | regulation of cytoskeleton organization |  | • |  |  |  |  |  |
| GO Bio Pro | cell-cell adhesion via plasma-membrane adhesion molecules |  | • |  |  |  |  |  |
| GO Bio Pro | microtubule-based process |  | • |  |  |  |  |  |
| GO Cell Comp | cell projection |  | • |  |  |  |  |  |
| GO Bio Pro | actin cytoskeleton organization |  | • |  |  |  |  |  |
| GO Bio Pro | negative chemotaxis |  | • |  |  |  |  |  |
| GO Bio Pro | cell projection morphogenesis |  | • |  |  |  |  |  |
| GO Bio Pro | positive regulation of locomotion |  | • |  |  |  |  |  |
| GO Bio Pro | negative regulation of locomotion |  | • |  |  |  |  |  |
| GO Mol Func | cell adhesion molecule binding |  | • |  |  |  |  |  |
| GO Bio Pro | ameboidal-type cell migration |  | • |  |  |  |  |  |
| GO Bio Pro | cell adhesion |  | • |  |  |  |  |  |
| WikiPathways | Regulation of Microtubule Cytoskeleton |  | • |  |  |  |  |  |
| GO Bio Pro | adherens junction assembly |  | • |  |  |  |  |  |
| GO Bio Pro | microtubule cytoskeleton organization |  | • |  |  |  |  |  |
| KEGG | Adherens junction |  | • |  |  |  |  |  |
| GO Bio Pro | regulation of cellular component movement |  | • |  |  |  |  |  |
| GO Bio Pro | regulation of actin filament organization |  | • |  |  |  |  |  |
| GO Bio Pro | regulation of cell size |  | • |  |  |  |  |  |
| GO Bio Pro | regulation of cell-matrix adhesion |  | • |  |  |  |  |  |
| WikiPathways | Cell migration and invasion through p75NTR |  | • |  |  |  |  |  |
| GO Bio Pro | positive regulation of cell adhesion |  | • |  |  |  |  |  |

Supplementary Table 3. Pathway Groups (cont.)

| Database | Pathway | Cancer | Motility | DNA Damage | Cell Cycle | Apoptosis | Miscellaneous | Canonical |
| --- | --- | --- | --- | --- | --- | --- | --- | --- |
| GO Bio Pro | regulation of cellular component size |  | • |  |  |  |  |  |
| GO Cell Comp | spindle microtubule |  | • |  |  |  |  |  |
| GO Bio Pro | adherens junction organization |  | • |  |  |  |  |  |
| GO Bio Pro | cell-substrate adhesion |  | • |  |  |  |  |  |
| GO Bio Pro | cell-cell adhesion |  | • |  |  |  |  |  |
| GO Bio Pro | cytoskeleton organization |  | • |  |  |  |  |  |
| GO Cell Comp | focal adhesion |  | • |  |  |  |  |  |
| GO Bio Pro | regulation of plasma membrane bounded cell projection organization |  | • |  |  |  |  |  |
| GO Bio Pro | positive regulation of cell projection organization |  | • |  |  |  |  |  |
| GO Bio Pro | regulation of cell-substrate adhesion |  | • |  |  |  |  |  |
| GO Bio Pro | actin cytoskeleton reorganization |  | • |  |  |  |  |  |
| GO Cell Comp | cell-substrate adherens junction |  | • |  |  |  |  |  |
| GO Bio Pro | regulation of cell projection organization |  | • |  |  |  |  |  |
| WikiPathways | Regulation of Actin Cytoskeleton |  | • |  |  |  |  |  |
| GO Mol Func | cadherin binding |  | • |  |  |  |  |  |
| GO Cell Comp | plasma membrane bounded cell projection |  | • |  |  |  |  |  |
| GO Bio Pro | cell projection organization |  | • |  |  |  |  |  |
| GO Cell Comp | lamellipodium |  | • |  |  |  |  |  |
| GO Bio Pro | positive regulation of cellular component movement |  | • |  |  |  |  |  |
| GO Mol Func | cytoskeletal protein binding |  | • |  |  |  |  |  |
| GO Bio Pro | regulation of actin cytoskeleton reorganization |  | • |  |  |  |  |  |
| GO Bio Pro | cortical cytoskeleton organization |  | • |  |  |  |  |  |
| GO Bio Pro | regulation of cell migration |  | • |  |  |  |  |  |
| GO Bio Pro | actin filament-based process |  | • |  |  |  |  |  |
| GO Bio Pro | homophilic cell adhesion via plasma membrane adhesion molecules |  | • |  |  |  |  |  |
| GO Bio Pro | lamellipodium morphogenesis |  | • |  |  |  |  |  |
| WikiPathways | Focal Adhesion |  | • |  |  |  |  |  |
| GO Bio Pro | positive regulation of cell migration |  | • |  |  |  |  |  |
| KEGG | Focal adhesion |  | • |  |  |  |  |  |
| GO Bio Pro | establishment or maintenance of cell polarity |  | • |  |  |  |  |  |
| GO Bio Pro | protein localization to cytoskeleton |  | • |  |  |  |  |  |
| GO Bio Pro | regulation of locomotion |  | • |  |  |  |  |  |

Supplementary Table 3. Pathway Groups (cont.)

| Database | Pathway | Cancer | Motility | DNA Damage | Cell Cycle | Apoptosis | Miscellaneous | Canonical |
| --- | --- | --- | --- | --- | --- | --- | --- | --- |
| GO Bio Pro | regulation of actin cytoskeleton organization |  | • |  |  |  |  |  |
| GO Bio Pro | locomotion |  | • |  |  |  |  |  |
| GO Cell Comp | filopodium |  | • |  |  |  |  |  |
| GO Bio Pro | cell-matrix adhesion |  | • |  |  |  |  |  |
| GO Cell Comp | cell leading edge |  | • |  |  |  |  |  |
| GO Cell Comp | adherens junction |  | • |  |  |  |  |  |
| GO Bio Pro | regulation of cell adhesion |  | • |  |  |  |  |  |
| GO Bio Pro | regulation of focal adhesion assembly |  | • |  |  |  |  |  |
| GO Bio Pro | focal adhesion assembly |  | • |  |  |  |  |  |
| GO Bio Pro | actin filament organization |  | • |  |  |  |  |  |
| GO Bio Pro | cell projection assembly |  | • |  |  |  |  |  |
| GO Bio Pro | establishment of cell polarity |  | • |  |  |  |  |  |
| Reactome | G1/S DNA Damage Checkpoints |  |  | • |  |  |  |  |
| WikiPathways | DNA Damage Response (only ATM dependent) |  |  | • |  |  |  |  |
| GO Mol Func | damaged DNA binding |  |  | • |  |  |  |  |
| GO Bio Pro | double-strand break repair |  |  | • |  |  |  |  |
| Reactome | DNA Repair |  |  | • |  |  |  |  |
| GO Bio Pro | G1 DNA damage checkpoint |  |  | • |  |  |  |  |
| GO Bio Pro | signal transduction in response to DNA damage |  |  | • |  |  |  |  |
| GO Bio Pro | regulation of DNA damage response, signal transduction by p53 class mediator |  |  | • |  |  |  |  |
| GO Bio Pro | nucleotide-excision repair, preincision complex assembly |  |  | • |  |  |  |  |
| Reactome | DNA Double-Strand Break Repair |  |  | • |  |  |  |  |
| WikiPathways | miRNA Regulation of DNA Damage Response |  |  | • |  |  |  |  |
| Reactome | Nucleotide Excision Repair |  |  | • |  |  |  |  |
| GO Bio Pro | signal transduction involved in DNA damage checkpoint |  |  | • |  |  |  |  |
| WikiPathways | DNA Damage Response |  |  | • |  |  |  |  |
| KEGG | Nucleotide excision repair |  |  | • |  |  |  |  |
| GO Bio Pro | regulation of DNA repair |  |  | • |  |  |  |  |
| GO Bio Pro | DNA damage checkpoint |  |  | • |  |  |  |  |
| GO Bio Pro | double-strand break repair via homologous recombination |  |  | • |  |  |  |  |

Supplementary Table 3. Pathway Groups (cont.)

| Database | Pathway | Cancer | Motility | DNA Damage | Cell Cycle | Apoptosis | Miscellaneous | Canonical |
| --- | --- | --- | --- | --- | --- | --- | --- | --- |
| Reactome | Global Genome Nucleotide Excision Repair (GG-NER) |  |  | • |  |  |  |  |
| GO Bio Pro | DNA repair |  |  | • |  |  |  |  |
| GO Bio Pro | positive regulation of response to DNA damage stimulus |  |  | • |  |  |  |  |
| GO Bio Pro | cellular response to DNA damage stimulus |  |  | • |  |  |  |  |
| Reactome | p53-Dependent G1 DNA Damage Response |  |  | • |  |  |  |  |
| GO Bio Pro | regulation of response to DNA damage stimulus |  |  | • |  |  |  |  |
| GO Bio Pro | DNA damage response, signal transduction by p53 class mediator resulting in cell cycle arrest |  |  | • |  |  |  |  |
| GO Bio Pro | recombinational repair |  |  | • |  |  |  |  |
| GO Bio Pro | nucleotide-excision repair, DNA damage recognition |  |  | • |  |  |  |  |
| GO Bio Pro | DNA damage response, signal transduction by p53 class mediator |  |  | • |  |  |  |  |
| Reactome | p53-Dependent G1/S DNA damage checkpoint |  |  | • |  |  |  |  |
| Reactome | SUMOylation of DNA damage response and repair proteins |  |  | • |  |  |  |  |
| GO Bio Pro | global genome nucleotide-excision repair |  |  | • |  |  |  |  |
| WikiPathways | miRNAs involved in DNA damage response |  |  | • |  |  |  |  |
| GO Bio Pro | mitotic G1 DNA damage checkpoint |  |  | • |  |  |  |  |
| Reactome | DNA Damage Recognition in GG-NER |  |  | • |  |  |  |  |
| GO Bio Pro | signal transduction involved in mitotic G1 DNA damage checkpoint |  |  | • |  |  |  |  |
| GO Cell Comp | site of DNA damage |  |  | • |  |  |  |  |
| Reactome | DNA Damage/Telomere Stress Induced Senescence |  |  | • |  |  |  |  |
| GO Bio Pro | regulation of cell growth |  |  |  | • |  |  |  |
| GO Bio Pro | regulation of cell cycle process |  |  |  | • |  |  |  |
| GO Bio Pro | cell cycle phase transition |  |  |  | • |  |  |  |
| GO Bio Pro | negative regulation of G1/S transition of mitotic cell cycle |  |  |  | • |  |  |  |
| Reactome | Mitotic G1-G1/S phases |  |  |  | • |  |  |  |
| GO Bio Pro | regulation of cell cycle |  |  |  | • |  |  |  |
| Reactome | Cell Cycle, Mitotic |  |  |  | • |  |  |  |

Supplementary Table 3. Pathway Groups (cont.)

| Database | Pathway | Cancer | Motility | DNA Damage | Cell Cycle | Apoptosis | Miscellaneous | Canonical |
| --- | --- | --- | --- | --- | --- | --- | --- | --- |
| GO Bio Pro | mitotic G1/S transition checkpoint |  |  |  | • |  |  |  |
| GO Bio Pro | mitotic nuclear division |  |  |  | • |  |  |  |
| GO Bio Pro | positive regulation of cell cycle arrest |  |  |  | • |  |  |  |
| GO Bio Pro | regulation of cell cycle phase transition |  |  |  | • |  |  |  |
| GO Bio Pro | cell cycle arrest |  |  |  | • |  |  |  |
| GO Bio Pro | regulation of G1/S transition of mitotic cell cycle |  |  |  | • |  |  |  |
| GO Bio Pro | positive regulation of cell cycle |  |  |  | • |  |  |  |
| GO Bio Pro | regulation of centrosome cycle |  |  |  | • |  |  |  |
| GO Bio Pro | homeostasis of number of cells within a tissue |  |  |  | • |  |  |  |
| GO Bio Pro | positive regulation of cell cycle G2/M phase transition |  |  |  | • |  |  |  |
| GO Bio Pro | G1/S transition of mitotic cell cycle |  |  |  | • |  |  |  |
| GO Bio Pro | negative regulation of cell growth |  |  |  | • |  |  |  |
| GO Bio Pro | regulation of cell cycle arrest |  |  |  | • |  |  |  |
| Reactome | Mitotic Prophase |  |  |  | • |  |  |  |
| GO Bio Pro | cell cycle |  |  |  | • |  |  |  |
| GO Bio Pro | positive regulation of cell cycle process |  |  |  | • |  |  |  |
| GO Bio Pro | mitotic cell cycle arrest |  |  |  | • |  |  |  |
| GO Cell Comp | replication fork |  |  |  | • |  |  |  |
| GO Bio Pro | homeostasis of number of cells |  |  |  | • |  |  |  |
| GO Bio Pro | negative regulation of mitotic cell cycle |  |  |  | • |  |  |  |
| GO Bio Pro | signal transduction involved in cell cycle checkpoint |  |  |  | • |  |  |  |
| GO Bio Pro | negative regulation of mitotic cell cycle phase transition |  |  |  | • |  |  |  |
| GO Bio Pro | positive regulation of mitotic cell cycle |  |  |  | • |  |  |  |
| GO Bio Pro | mitotic cell cycle phase transition |  |  |  | • |  |  |  |
| GO Bio Pro | regulation of mitotic cell cycle phase transition |  |  |  | • |  |  |  |
| GO Bio Pro | DNA-dependent DNA replication |  |  |  | • |  |  |  |
| GO Bio Pro | regulation of mitotic cell cycle |  |  |  | • |  |  |  |
| GO Bio Pro | regulation of cell cycle G1/S phase transition |  |  |  | • |  |  |  |
| KEGG | Cell cycle |  |  |  | • |  |  |  |
| Reactome | Cell Cycle |  |  |  | • |  |  |  |
| WikiPathways | G1 to S cell cycle control |  |  |  | • |  |  |  |
| GO Bio Pro | mitotic sister chromatid segregation |  |  |  | • |  |  |  |

Supplementary Table 3. Pathway Groups (cont.)

| Database | Pathway | Cancer | Motility | DNA Damage | Cell Cycle | Apoptosis | Miscellaneous | Canonical |
| --- | --- | --- | --- | --- | --- | --- | --- | --- |
| GO Bio Pro | negative regulation of cell cycle process |  |  |  | • |  |  |  |
| GO Bio Pro | negative regulation of cell cycle G1/S phase transition |  |  |  | • |  |  |  |
| GO Bio Pro | regulation of mitotic spindle assembly |  |  |  | • |  |  |  |
| GO Bio Pro | cell cycle G1/S phase transition |  |  |  | • |  |  |  |
| GO Bio Pro | positive regulation of cell cycle phase transition |  |  |  | • |  |  |  |
| GO Bio Pro | DNA replication |  |  |  | • |  |  |  |
| GO Bio Pro | mitotic cell cycle |  |  |  | • |  |  |  |
| GO Bio Pro | mitotic sister chromatid cohesion |  |  |  | • |  |  |  |
| WikiPathways | Cell Cycle |  |  |  | • |  |  |  |
| GO Bio Pro | negative regulation of cell cycle |  |  |  | • |  |  |  |
| GO Bio Pro | regulation of oxidative stress-induced intrinsic apoptotic signaling pathway |  |  |  |  | • |  |  |
| GO Bio Pro | intrinsic apoptotic signaling pathway in response to DNA damage by p53 class mediator |  |  |  |  | • |  |  |
| GO Bio Pro | regulation of extrinsic apoptotic signaling pathway |  |  |  |  | • |  |  |
| GO Bio Pro | regulation of intrinsic apoptotic signaling pathway by p53 class mediator |  |  |  |  | • |  |  |
| WikiPathways | Apoptosis Modulation and Signaling |  |  |  |  | • |  |  |
| GO Bio Pro | intrinsic apoptotic signaling pathway |  |  |  |  | • |  |  |
| GO Bio Pro | extrinsic apoptotic signaling pathway via death domain receptors |  |  |  |  | • |  |  |
| GO Bio Pro | regulation of extrinsic apoptotic signaling pathway in absence of ligand |  |  |  |  | • |  |  |
| GO Bio Pro | intrinsic apoptotic signaling pathway in response to endoplasmic reticulum stress |  |  |  |  | • |  |  |
| GO Bio Pro | protein insertion into mitochondrial membrane involved in apoptotic signaling pathway |  |  |  |  | • |  |  |
| GO Bio Pro | extrinsic apoptotic signaling pathway in absence of ligand |  |  |  |  | • |  |  |
| Reactome | Apoptosis |  |  |  |  | • |  |  |
| GO Bio Pro | negative regulation of extrinsic apoptotic signaling pathway in absence of ligand |  |  |  |  | • |  |  |
| Reactome | Formation of apoptosome |  |  |  |  | • |  |  |
| GO Bio Pro | apoptotic DNA fragmentation |  |  |  |  | • |  |  |
| Reactome | Apoptotic factor-mediated response |  |  |  |  | • |  |  |

Supplementary Table 3. Pathway Groups (cont.)

| Database | Pathway | Cancer | Motility | DNA Damage | Cell Cycle | Apoptosis | Miscellaneous | Canonical |
| --- | --- | --- | --- | --- | --- | --- | --- | --- |
| Reactome | Cytochrome c-mediated apoptotic response |  |  |  |  | • |  |  |
| GO Bio Pro | intrinsic apoptotic signaling pathway in response to oxidative stress |  |  |  |  | • |  |  |
| GO Bio Pro | apoptotic signaling pathway |  |  |  |  | • |  |  |
| GO Bio Pro | extrinsic apoptotic signaling pathway |  |  |  |  | • |  |  |
| GO Bio Pro | apoptotic process involved in morphogenesis |  |  |  |  | • |  |  |
| GO Bio Pro | regulation of intrinsic apoptotic signaling pathway |  |  |  |  | • |  |  |
| GO Bio Pro | positive regulation of apoptotic process |  |  |  |  | • |  |  |
| GO Bio Pro | positive regulation of intrinsic apoptotic signaling pathway |  |  |  |  | • |  |  |
| KEGG | Apoptosis |  |  |  |  | • |  |  |
| WikiPathways | Apoptosis |  |  |  |  | • |  |  |
| GO Bio Pro | negative regulation of apoptotic process |  |  |  |  | • |  |  |
| GO Bio Pro | negative regulation of intrinsic apoptotic signaling pathway by p53 class mediator |  |  |  |  | • |  |  |
| Reactome | Regulation of the apoptosome activity |  |  |  |  | • |  |  |
| Reactome | Intrinsic Pathway for Apoptosis |  |  |  |  | • |  |  |
| GO Bio Pro | regulation of apoptotic process |  |  |  |  | • |  |  |
| GO Bio Pro | apoptotic process |  |  |  |  | • |  |  |
| GO Bio Pro | positive regulation of apoptotic signaling pathway |  |  |  |  | • |  |  |
| GO Bio Pro | positive regulation of endoplasmic reticulum stress-induced intrinsic apoptotic signaling pathway |  |  |  |  | • |  |  |
| GO Bio Pro | intrinsic apoptotic signaling pathway in response to DNA damage |  |  |  |  | • |  |  |
| GO Bio Pro | intrinsic apoptotic signaling pathway by p53 class mediator |  |  |  |  | • |  |  |
| GO Bio Pro | negative regulation of extrinsic apoptotic signaling pathway |  |  |  |  | • |  |  |
| GO Bio Pro | negative regulation of apoptotic signaling pathway |  |  |  |  | • |  |  |
| GO Bio Pro | regulation of endoplasmic reticulum stress-induced intrinsic apoptotic signaling pathway |  |  |  |  | • |  |  |
| GO Cell Comp | RISC complex |  |  |  |  |  | • |  |
| GO Bio Pro | miRNA mediated inhibition of translation |  |  |  |  |  | • |  |
| GO Bio Pro | miRNA metabolic process |  |  |  |  |  | • |  |

Supplementary Table 3. Pathway Groups (cont.)

| Database | Pathway | Cancer | Motility | DNA Damage | Cell Cycle | Apoptosis | Miscellaneous | Canonical |
| --- | --- | --- | --- | --- | --- | --- | --- | --- |
| GO Bio Pro | cellular response to mechanical stimulus |  |  |  |  |  | • |  |
| WikiPathways | MAPK Cascade |  |  |  |  |  |  | • |
| Reactome | Constitutive Signaling by Aberrant PI3K in Cancer |  |  |  |  |  |  | • |
| GO Bio Pro | regulation of Notch signaling pathway |  |  |  |  |  |  | • |
| WikiPathways | IL-4 Signaling Pathway |  |  |  |  |  |  | • |
| KEGG | Hedgehog signaling pathway |  |  |  |  |  |  | • |
| GO Bio Pro | hippo signaling |  |  |  |  |  |  | • |
| Reactome | RAF/MAP kinase cascade |  |  |  |  |  |  | • |
| Reactome | Cytokine Signaling in Immune system |  |  |  |  |  |  | • |
| Reactome | MAPK1/MAPK3 signaling |  |  |  |  |  |  | • |
| WikiPathways | RANKL/RANK (Receptor activator of NFKB (ligand)) Signaling Pathway |  |  |  |  |  |  | • |
| WikiPathways | ATM Signaling Network in Development and Disease |  |  |  |  |  |  | • |
| GO Bio Pro | regulation of canonical Wnt signaling pathway |  |  |  |  |  |  | • |
| WikiPathways | TGF-beta Signaling Pathway |  |  |  |  |  |  | • |
| GO Bio Pro | Fc-gamma receptor signaling pathway |  |  |  |  |  |  | • |
| GO Bio Pro | transmembrane receptor protein tyrosine kinase signaling pathway |  |  |  |  |  |  | • |
| GO Bio Pro | lipopolysaccharide-mediated signaling pathway |  |  |  |  |  |  | • |
| GO Bio Pro | intracellular estrogen receptor signaling pathway |  |  |  |  |  |  | • |
| Reactome | Interleukin-4 and Interleukin-13 signaling |  |  |  |  |  |  | • |
| Reactome | Signaling by EGFRvIII in Cancer |  |  |  |  |  |  | • |
| Reactome | CREB1 phosphorylation through NMDA receptor-mediated activation of RAS signaling |  |  |  |  |  |  | • |
| KEGG | Chemokine signaling pathway |  |  |  |  |  |  | • |
| GO Bio Pro | cell-cell signaling |  |  |  |  |  |  | • |
| KEGG | Prolactin signaling pathway |  |  |  |  |  |  | • |
| Reactome | SHC-mediated cascade:FGFR1 |  |  |  |  |  |  | • |
| Reactome | Signaling by FGFR2 in disease |  |  |  |  |  |  | • |
| Reactome | Netrin-1 signaling |  |  |  |  |  |  | • |
| GO Bio Pro | positive regulation of signaling receptor activity |  |  |  |  |  |  | • |
| WikiPathways | TGF-beta Receptor Signaling |  |  |  |  |  |  | • |

Supplementary Table 3. Pathway Groups (cont.)

| Database | Pathway | Cancer | Motility | DNA Damage | Cell Cycle | Apoptosis | Miscellaneous | Canonical |
| --- | --- | --- | --- | --- | --- | --- | --- | --- |
| WikiPathways | Focal Adhesion-PI3K-Akt-mTOR-signaling pathway |  |  |  |  |  |  | • |
| GO Bio Pro | BMP signaling pathway |  |  |  |  |  |  | • |
| WikiPathways | Corticotropin-releasing hormone signaling pathway |  |  |  |  |  |  | • |
| WikiPathways | IL-2 Signaling Pathway |  |  |  |  |  |  | • |
| Reactome | Signaling by FGFR in disease |  |  |  |  |  |  | • |
| Reactome | Negative regulation of MAPK pathway |  |  |  |  |  |  | • |
| WikiPathways | Thymic Stromal LymphoPoietin (TSLP) Signaling Pathway |  |  |  |  |  |  | • |
| WikiPathways | Leptin signaling pathway |  |  |  |  |  |  | • |
| WikiPathways | Notch Signaling Pathway |  |  |  |  |  |  | • |
| WikiPathways | TNF alpha Signaling Pathway |  |  |  |  |  |  | • |
| Reactome | Toll Like Receptor 4 (TLR4) Cascade |  |  |  |  |  |  | • |
| WikiPathways | PI3K-Akt Signaling Pathway |  |  |  |  |  |  | • |
| GO Bio Pro | cell-cell signaling by wnt |  |  |  |  |  |  | • |
| KEGG | Phosphatidylinositol signaling system |  |  |  |  |  |  | • |
| Reactome | Paradoxical activation of RAF signaling by kinase inactive BRAF |  |  |  |  |  |  | • |
| Reactome | Neurotransmitter receptors and postsynaptic signal transmission |  |  |  |  |  |  | • |
| GO Bio Pro | regulation of Wnt signaling pathway |  |  |  |  |  |  | • |
| Reactome | SHC-mediated cascade:FGFR2 |  |  |  |  |  |  | • |
| WikiPathways | H19 action Rb-E2F1 signaling and CDK-Beta-catenin activity |  |  |  |  |  |  | • |
| GO Bio Pro | regulation of androgen receptor signaling pathway |  |  |  |  |  |  | • |
| GO Mol Func | nuclear receptor transcription coactivator activity |  |  |  |  |  |  | • |
| Reactome | Signaling by PTK6 |  |  |  |  |  |  | • |
| WikiPathways | G Protein Signaling Pathways |  |  |  |  |  |  | • |
| Reactome | Toll Like Receptor TLR6:TLR2 Cascade |  |  |  |  |  |  | • |
| WikiPathways | Serotonin Receptor 2 and ELK-SRF/GATA4 signaling |  |  |  |  |  |  | • |
| WikiPathways | Estrogen signaling pathway |  |  |  |  |  |  | • |
| KEGG | TNF signaling pathway |  |  |  |  |  |  | • |
| Reactome | Signaling by Type 1 Insulin-like Growth Factor 1 Receptor (IGF1R) |  |  |  |  |  |  | • |
| GO Bio Pro | regulation of signaling |  |  |  |  |  |  | • |

Supplementary Table 3. Pathway Groups (cont.)

| Database | Pathway | Cancer | Motility | DNA Damage | Cell Cycle | Apoptosis | Miscellaneous | Canonical |
| --- | --- | --- | --- | --- | --- | --- | --- | --- |
| WikiPathways | PDGF Pathway |  |  |  |  |  |  | • |
| WikiPathways | Alpha 6 Beta 4 signaling pathway |  |  |  |  |  |  | • |
| Reactome | Extra-nuclear estrogen signaling |  |  |  |  |  |  | • |
| GO Bio Pro | Wnt signaling pathway |  |  |  |  |  |  | • |
| Reactome | Signaling by Non-Receptor Tyrosine Kinases |  |  |  |  |  |  | • |
| KEGG | Fc epsilon RI signaling pathway |  |  |  |  |  |  | • |
| WikiPathways | Type II interferon signaling (IFNG) |  |  |  |  |  |  | • |
| GO Bio Pro | positive regulation of transforming growth factor beta receptor signaling pathway |  |  |  |  |  |  | • |
| GO Bio Pro | Notch signaling involved in heart development |  |  |  |  |  |  | • |
| GO Bio Pro | negative regulation of protein kinase B signaling |  |  |  |  |  |  | • |
| Reactome | Signaling by Erythropoietin |  |  |  |  |  |  | • |
| WikiPathways | VEGFA-VEGFR2 Signaling Pathway |  |  |  |  |  |  | • |
| GO Bio Pro | positive regulation of ERK1 and ERK2 cascade |  |  |  |  |  |  | • |
| WikiPathways | Notch Signaling |  |  |  |  |  |  | • |
| GO Bio Pro | activation of MAPK activity |  |  |  |  |  |  | • |
| KEGG | Insulin signaling pathway |  |  |  |  |  |  | • |
| GO Bio Pro | regulation of intracellular steroid hormone receptor signaling pathway |  |  |  |  |  |  | • |
| WikiPathways | IL-6 signaling pathway |  |  |  |  |  |  | • |
| GO Bio Pro | immune response-regulating signaling pathway |  |  |  |  |  |  | • |
| WikiPathways | Wnt Signaling Pathway |  |  |  |  |  |  | • |
| Reactome | Signaling by SCF-KIT |  |  |  |  |  |  | • |
| Reactome | SHC1 events in ERBB2 signaling |  |  |  |  |  |  | • |
| Reactome | Signaling by FGFR1 |  |  |  |  |  |  | • |
| WikiPathways | Synaptic signaling pathways associated with autism spectrum disorder |  |  |  |  |  |  | • |
| KEGG | MAPK signaling pathway |  |  |  |  |  |  | • |
| Reactome | Downstream signaling of activated FGFR1 |  |  |  |  |  |  | • |
| Reactome | NCAM signaling for neurite out-growth |  |  |  |  |  |  | • |
| GO Bio Pro | I-kappaB kinase/NF-kappaB signaling |  |  |  |  |  |  | • |
| KEGG | Ras signaling pathway |  |  |  |  |  |  | • |

Supplementary Table 3. Pathway Groups (cont.)

| Database | Pathway | Cancer | Motility | DNA Damage | Cell Cycle | Apoptosis | Miscellaneous | Canonical |
| --- | --- | --- | --- | --- | --- | --- | --- | --- |
| GO Bio Pro | cellular response to transforming growth factor beta stimulus |  |  |  |  |  |  | • |
| Reactome | IGF1R signaling cascade |  |  |  |  |  |  | • |
| WikiPathways | Brain-Derived Neurotrophic Factor (BDNF) signaling pathway |  |  |  |  |  |  | • |
| Reactome | Interleukin-12 signaling |  |  |  |  |  |  | • |
| Reactome | Downstream signaling of activated FGFR4 |  |  |  |  |  |  | • |
| GO Bio Pro | positive regulation of toll-like receptor 4 signaling pathway |  |  |  |  |  |  | • |
| GO Bio Pro | negative regulation of transforming growth factor beta1 production |  |  |  |  |  |  | • |
| GO Bio Pro | regulation of nucleotide-binding oligomerization domain containing signaling pathway |  |  |  |  |  |  | • |
| KEGG | Hippo signaling pathway |  |  |  |  |  |  | • |
| GO Bio Pro | MAPK cascade |  |  |  |  |  |  | • |
| Reactome | Intracellular signaling by second messengers |  |  |  |  |  |  | • |
| KEGG | Adipocytokine signaling pathway |  |  |  |  |  |  | • |
| GO Mol Func | transforming growth factor beta receptor binding |  |  |  |  |  |  | • |
| Reactome | GRB2 events in EGFR signaling |  |  |  |  |  |  | • |
| WikiPathways | Serotonin Receptor 4/6/7 and NR3C Signaling |  |  |  |  |  |  | • |
| GO Bio Pro | cellular response to growth factor stimulus |  |  |  |  |  |  | • |
| GO Bio Pro | JNK cascade |  |  |  |  |  |  | • |
| WikiPathways | Kit receptor signaling pathway |  |  |  |  |  |  | • |
| GO Mol Func | MAP kinase activity |  |  |  |  |  |  | • |
| GO Bio Pro | androgen receptor signaling pathway |  |  |  |  |  |  | • |
| KEGG | Estrogen signaling pathway |  |  |  |  |  |  | • |
| WikiPathways | Androgen receptor signaling pathway |  |  |  |  |  |  | • |
| GO Bio Pro | platelet-derived growth factor receptor signaling pathway |  |  |  |  |  |  | • |
| GO Bio Pro | interleukin-6-mediated signaling pathway |  |  |  |  |  |  | • |
| WikiPathways | Human Thyroid Stimulating Hormone (TSH) signaling pathway |  |  |  |  |  |  | • |
| Reactome | Signaling by NOTCH1 in Cancer |  |  |  |  |  |  | • |
| Reactome | MAPK family signaling cascades |  |  |  |  |  |  | • |
| WikiPathways | Pathways Regulating Hippo Signaling |  |  |  |  |  |  | • |

Supplementary Table 3. Pathway Groups (cont.)

| Database | Pathway | Cancer | Motility | DNA Damage | Cell Cycle | Apoptosis | Miscellaneous | Canonical |
| --- | --- | --- | --- | --- | --- | --- | --- | --- |
| GO Bio Pro | cell surface receptor signaling pathway |  |  |  |  |  |  | • |
| Reactome | Signaling by TGF-beta Receptor Complex |  |  |  |  |  |  | • |
| WikiPathways | RAC1/PAK1/p38/MMP2 Pathway |  |  |  |  |  |  | • |
| Reactome | DAP12 signaling |  |  |  |  |  |  | • |
| Reactome | Other interleukin signaling |  |  |  |  |  |  | • |
| KEGG | TGF-beta signaling pathway |  |  |  |  |  |  | • |
| GO Bio Pro | positive regulation of MAPK cascade |  |  |  |  |  |  | • |
| GO Bio Pro | response to transforming growth factor beta |  |  |  |  |  |  | • |
| Reactome | VEGFA-VEGFR2 Pathway |  |  |  |  |  |  | • |
| Reactome | Signaling by ERBB4 |  |  |  |  |  |  | • |
| WikiPathways | IL-3 Signaling Pathway |  |  |  |  |  |  | • |
| WikiPathways | Interleukin-11 Signaling Pathway |  |  |  |  |  |  | • |
| Reactome | SHC1 events in ERBB4 signaling |  |  |  |  |  |  | • |
| GO Bio Pro | transforming growth factor beta receptor signaling pathway |  |  |  |  |  |  | • |
| Reactome | MAPK targets/ Nuclear events mediated by MAP kinases |  |  |  |  |  |  | • |
| Reactome | Fc epsilon receptor (FCERI) signaling |  |  |  |  |  |  | • |
| WikiPathways | PI3K-AKT-mTOR signaling pathway and therapeutic opportunities |  |  |  |  |  |  | • |
| Reactome | MET activates RAS signaling |  |  |  |  |  |  | • |
| Reactome | Signaling by Hippo |  |  |  |  |  |  | • |
| Reactome | Signaling by ERBB2 |  |  |  |  |  |  | • |
| Reactome | Signaling by VEGF |  |  |  |  |  |  | • |
| GO Bio Pro | intracellular steroid hormone receptor signaling pathway |  |  |  |  |  |  | • |
| WikiPathways | IL-9 Signaling Pathway |  |  |  |  |  |  | • |
| Reactome | SHC-related events triggered by IGF1R |  |  |  |  |  |  | • |
| Reactome | Signaling by MET |  |  |  |  |  |  | • |
| Reactome | Signaling by NOTCH1 |  |  |  |  |  |  | • |
| Reactome | Downstream signaling of activated FGFR3 |  |  |  |  |  |  | • |
| Reactome | Signaling by TGF-beta Receptor Complex in Cancer |  |  |  |  |  |  | • |
| Reactome | FLT3 Signaling |  |  |  |  |  |  | • |
| Reactome | Interferon Signaling |  |  |  |  |  |  | • |
| Reactome | Post NMDA receptor activation events |  |  |  |  |  |  | • |

Supplementary Table 3. Pathway Groups (cont.)

| Database | Pathway | Cancer | Motility | DNA Damage | Cell Cycle | Apoptosis | Miscellaneous | Canonical |
| --- | --- | --- | --- | --- | --- | --- | --- | --- |
| GO Bio Pro | hormone-mediated signaling pathway |  |  |  |  |  |  | • |
| Reactome | Interleukin-6 signaling |  |  |  |  |  |  | • |
| KEGG | Rap1 signaling pathway |  |  |  |  |  |  | • |
| Reactome | Regulation of TP53 Activity |  |  |  |  |  |  | • |
| WikiPathways | LncRNA involvement in canonical Wnt signaling and colorectal cancer |  |  |  |  |  |  | • |
| GO Bio Pro | cell surface receptor signaling pathway involved in cell-cell signaling |  |  |  |  |  |  | • |
| Reactome | Signaling by BRAF and RAF fusions |  |  |  |  |  |  | • |
| Reactome | Signaling by FGFR4 in disease |  |  |  |  |  |  | • |
| Reactome | Signaling by BMP |  |  |  |  |  |  | • |
| GO Bio Pro | Notch signaling pathway |  |  |  |  |  |  | • |
| GO Mol Func | nuclear hormone receptor binding |  |  |  |  |  |  | • |
| GO Bio Pro | Fc receptor mediated stimulatory signaling pathway |  |  |  |  |  |  | • |
| GO Bio Pro | transmembrane receptor protein serine/threonine kinase signaling pathway |  |  |  |  |  |  | • |
| WikiPathways | Prolactin Signaling Pathway |  |  |  |  |  |  | • |
| WikiPathways | Wnt Signaling Pathway and Pluripotency |  |  |  |  |  |  | • |
| Reactome | Signaling by Leptin |  |  |  |  |  |  | • |
| Reactome | Oncogenic MAPK signaling |  |  |  |  |  |  | • |
| Reactome | Signaling by PDGF |  |  |  |  |  |  | • |
| Reactome | Toll Like Receptor 2 (TLR2) Cascade |  |  |  |  |  |  | • |
| GO Bio Pro | ER-nucleus signaling pathway |  |  |  |  |  |  | • |
| Reactome | Estrogen-stimulated signaling through PRKCZ |  |  |  |  |  |  | • |
| Reactome | FRS-mediated FGFR4 signaling |  |  |  |  |  |  | • |
| GO Bio Pro | positive regulation of I-kappaB kinase/NF-kappaB signaling |  |  |  |  |  |  | • |
| KEGG | VEGF signaling pathway |  |  |  |  |  |  | • |
| WikiPathways | TP53 Network |  |  |  |  |  |  | • |
| GO Bio Pro | stress-activated MAPK cascade |  |  |  |  |  |  | • |
| Reactome | GRB2 events in ERBB2 signaling |  |  |  |  |  |  | • |
| Reactome | FRS-mediated FGFR2 signaling |  |  |  |  |  |  | • |
| Reactome | Signaling by TGF-beta family members |  |  |  |  |  |  | • |
| GO Bio Pro | positive regulation of Notch signaling pathway |  |  |  |  |  |  | • |

Supplementary Table 3. Pathway Groups (cont.)

| Database | Pathway | Cancer | Motility | DNA Damage | Cell Cycle | Apoptosis | Miscellaneous | Canonical |
| --- | --- | --- | --- | --- | --- | --- | --- | --- |
| Reactome | PI5P, PP2A and IER3 Regulate PI3K/AKT Signaling |  |  |  |  |  |  | • |
| GO Bio Pro | steroid hormone mediated signaling pathway |  |  |  |  |  |  | • |
| Reactome | Signaling by NTRKs |  |  |  |  |  |  | • |
| KEGG | Neurotrophin signaling pathway |  |  |  |  |  |  | • |
| WikiPathways | ATM Signaling Pathway |  |  |  |  |  |  | • |
| GO Bio Pro | activin receptor signaling pathway |  |  |  |  |  |  | • |
| Reactome | MyD88:MAL(TIRAP) cascade initiated on plasma membrane |  |  |  |  |  |  | • |
| Reactome | Downstream signaling of activated FGFR2 |  |  |  |  |  |  | • |
| Reactome | Growth hormone receptor signaling |  |  |  |  |  |  | • |
| Reactome | FRS-mediated FGFR1 signaling |  |  |  |  |  |  | • |
| Reactome | Signaling by NTRK2 (TRKB) |  |  |  |  |  |  | • |
| Reactome | ESR-mediated signaling |  |  |  |  |  |  | • |
| GO Bio Pro | Wnt signaling pathway, calcium modulating pathway |  |  |  |  |  |  | • |
| Reactome | SHC-mediated cascade:FGFR4 |  |  |  |  |  |  | • |
| Reactome | Signaling by NTRK1 (TRKA) |  |  |  |  |  |  | • |
| Reactome | Signaling by WNT |  |  |  |  |  |  | • |
| KEGG | p53 signaling pathway |  |  |  |  |  |  | • |
| Reactome | Signaling by Interleukins |  |  |  |  |  |  | • |
| GO Bio Pro | ERK1 and ERK2 cascade |  |  |  |  |  |  | • |
| GO Bio Pro | leptin-mediated signaling pathway |  |  |  |  |  |  | • |
| Reactome | PIP3 activates AKT signaling |  |  |  |  |  |  | • |
| WikiPathways | Oncostatin M Signaling Pathway |  |  |  |  |  |  | • |
| GO Bio Pro | negative regulation of phosphatidylinositol 3-kinase signaling |  |  |  |  |  |  | • |
| Reactome | Signaling by FGFR3 in disease |  |  |  |  |  |  | • |
| WikiPathways | IL-1 signaling pathway |  |  |  |  |  |  | • |
| Reactome | Signaling by Receptor Tyrosine Kinases |  |  |  |  |  |  | • |
| WikiPathways | BDNF-TrkB Signaling |  |  |  |  |  |  | • |
| GO Bio Pro | regulation of intracellular estrogen receptor signaling pathway |  |  |  |  |  |  | • |
| WikiPathways | IL-5 Signaling Pathway |  |  |  |  |  |  | • |
| Reactome | PI3K events in ERBB2 signaling |  |  |  |  |  |  | • |
| GO Bio Pro | negative regulation of intracellular estrogen receptor signaling pathway |  |  |  |  |  |  | • |
| WikiPathways | G13 Signaling Pathway |  |  |  |  |  |  | • |

Supplementary Table 3. Pathway Groups (cont.)

| Database | Pathway | Cancer | Motility | DNA Damage | Cell Cycle | Apoptosis | Miscellaneous | Canonical |
| --- | --- | --- | --- | --- | --- | --- | --- | --- |
| WikiPathways | EPO Receptor Signaling |  |  |  |  |  |  | • |
| Reactome | Signaling by NOTCH |  |  |  |  |  |  | • |
| Reactome | Negative regulation of the PI3K/AKT network |  |  |  |  |  |  | • |
| Reactome | Signaling by FGFR1 in disease |  |  |  |  |  |  | • |
| GO Bio Pro | response to growth factor |  |  |  |  |  |  | • |
| KEGG | FoxO signaling pathway |  |  |  |  |  |  | • |
| Reactome | Signaling by FGFR |  |  |  |  |  |  | • |
| Reactome | SHC1 events in EGFR signaling |  |  |  |  |  |  | • |
| GO Bio Pro | cellular response to fibroblast growth factor stimulus |  |  |  |  |  |  | • |
| GO Bio Pro | nucleotide-binding oligomerization domain containing signaling pathway |  |  |  |  |  |  | • |
| Reactome | Estrogen-dependent nuclear events downstream of ESR-membrane signaling |  |  |  |  |  |  | • |
| GO Bio Pro | cellular response to vascular endothelial growth factor stimulus |  |  |  |  |  |  | • |
| KEGG | Wnt signaling pathway |  |  |  |  |  |  | • |
| Reactome | Toll Like Receptor TLR1:TLR2 Cascade |  |  |  |  |  |  | • |
| Reactome | Signaling by NTRK3 (TRKC) |  |  |  |  |  |  | • |
| GO Bio Pro | regulation of TOR signaling |  |  |  |  |  |  | • |
| GO Mol Func | androgen receptor binding |  |  |  |  |  |  | • |
| Reactome | Signaling by FGFR2 |  |  |  |  |  |  | • |
| KEGG | GnRH signaling pathway |  |  |  |  |  |  | • |
| WikiPathways | Ras Signaling |  |  |  |  |  |  | • |
| GO Bio Pro | regulation of stress-activated protein kinase signaling cascade |  |  |  |  |  |  | • |
| GO Bio Pro | signaling |  |  |  |  |  |  | • |
| Reactome | Death Receptor Signalling |  |  |  |  |  |  | • |
| GO Bio Pro | response to nerve growth factor |  |  |  |  |  |  | • |
| Reactome | Regulation of lipid metabolism by Peroxisome proliferator-activated receptor alpha (PPARalpha) |  |  |  |  |  |  | • |
| Reactome | CD209 (DC-SIGN) signaling |  |  |  |  |  |  | • |
| WikiPathways | Aryl Hydrocarbon Receptor Pathway |  |  |  |  |  |  | • |
| GO Bio Pro | regulation of transforming growth factor beta receptor signaling pathway |  |  |  |  |  |  | • |
| WikiPathways | Signaling of Hepatocyte Growth Factor Receptor |  |  |  |  |  |  | • |

Supplementary Table 3. Pathway Groups (cont.)

| Database | Pathway | Cancer | Motility | DNA Damage | Cell Cycle | Apoptosis | Miscellaneous | Canonical |
| --- | --- | --- | --- | --- | --- | --- | --- | --- |
| GO Bio Pro | negative regulation of transmembrane receptor protein serine/threonine kinase signaling pathway |  |  |  |  |  |  | • |
| GO Bio Pro | regulation of MAPK cascade |  |  |  |  |  |  | • |
| GO Mol Func | nuclear receptor binding |  |  |  |  |  |  | • |
| GO Bio Pro | immune response-activating cell surface receptor signaling pathway |  |  |  |  |  |  | • |
| WikiPathways | MAPK Signaling Pathway |  |  |  |  |  |  | • |
| GO Bio Pro | positive regulation of stress-activated protein kinase signaling cascade |  |  |  |  |  |  | • |
| GO Bio Pro | stress-activated protein kinase signaling cascade |  |  |  |  |  |  | • |
| GO Mol Func | growth factor receptor binding |  |  |  |  |  |  | • |
| WikiPathways | Wnt Signaling |  |  |  |  |  |  | • |
| GO Bio Pro | cytokine-mediated signaling pathway |  |  |  |  |  |  | • |
| GO Bio Pro | cytoplasmic pattern recognition receptor signaling pathway |  |  |  |  |  |  | • |
| KEGG | ErbB signaling pathway |  |  |  |  |  |  | • |
| WikiPathways | ErbB Signaling Pathway |  |  |  |  |  |  | • |
| GO Bio Pro | positive regulation of Wnt signaling pathway |  |  |  |  |  |  | • |
| WikiPathways | Interferon type I signaling pathways |  |  |  |  |  |  | • |
| GO Bio Pro | regulation of stress-activated MAPK cascade |  |  |  |  |  |  | • |
| GO Bio Pro | Fc receptor signaling pathway |  |  |  |  |  |  | • |
| WikiPathways | PDGFR-beta pathway |  |  |  |  |  |  | • |
| Reactome | p38MAPK events |  |  |  |  |  |  | • |
| GO Bio Pro | response to epidermal growth factor |  |  |  |  |  |  | • |
| GO Bio Pro | regulation of I-kappaB kinase/NF-kappaB signaling |  |  |  |  |  |  | • |
| KEGG | HIF-1 signaling pathway |  |  |  |  |  |  | • |
| GO Bio Pro | regulation of MAP kinase activity |  |  |  |  |  |  | • |
| GO Bio Pro | negative regulation of TOR signaling |  |  |  |  |  |  |  |
| WikiPathways | Chemokine signaling pathway |  |  |  |  |  |  |  |
| Reactome | Signaling by FGFR3 |  |  |  |  |  |  |  |
| GO Bio Pro | positive regulation of tumor necrosis factor-mediated signaling pathway |  |  |  |  |  |  |  |
| Reactome | Spry regulation of FGF signaling |  |  |  |  |  |  |  |
| Reactome | Tie2 Signaling |  |  |  |  |  |  |  |

Supplementary Table 3. Pathway Groups (cont.)

| Database | Pathway | Cancer | Motility | DNA Damage | Cell Cycle | Apoptosis | Miscellaneous | Canonical |
| --- | --- | --- | --- | --- | --- | --- | --- | --- |
| KEGG | Thyroid hormone signaling pathway |  |  |  |  |  |  |  |
| GO Mol Func | transmembrane receptor protein serine/threonine kinase activity |  |  |  |  |  |  |  |
| WikiPathways | PTF1A related regulatory pathway |  |  |  |  |  |  |  |
| GO Bio Pro | positive regulation of stress-activated MAPK cascade |  |  |  |  |  |  |  |
| Reactome | Constitutive Signaling by EGFRvIII |  |  |  |  |  |  |  |
| Reactome | TCF dependent signaling in response to WNT |  |  |  |  |  |  |  |
| Reactome | Activation of NMDA receptors and postsynaptic events |  |  |  |  |  |  |  |
| Reactome | Signaling by FGFR3 fusions in cancer |  |  |  |  |  |  |  |
| Reactome | RUNX3 regulates NOTCH signaling |  |  |  |  |  |  |  |
| GO Bio Pro | glutamate receptor signaling pathway |  |  |  |  |  |  |  |
| Reactome | SHC-mediated cascade:FGFR3 |  |  |  |  |  |  |  |
| GO Bio Pro | TOR signaling |  |  |  |  |  |  |  |
| GO Bio Pro | nucleotide-binding domain, leucine rich repeat containing receptor signaling pathway |  |  |  |  |  |  |  |
| GO Bio Pro | positive regulation of MAP kinase activity |  |  |  |  |  |  |  |
| GO Bio Pro | cellular response to epidermal growth factor stimulus |  |  |  |  |  |  |  |
| WikiPathways | Hippo-Merlin Signaling Dysregulation |  |  |  |  |  |  |  |
| Reactome | PI3K/AKT Signaling in Cancer |  |  |  |  |  |  |  |
| Reactome | Signaling by EGFR in Cancer |  |  |  |  |  |  |  |
| Reactome | Insulin receptor signalling cascade |  |  |  |  |  |  |  |
| GO Bio Pro | regulation of ERK1 and ERK2 cascade |  |  |  |  |  |  |  |
| GO Mol Func | hormone receptor binding |  |  |  |  |  |  | • |
| Reactome | Signaling by EGFR |  |  |  |  |  |  | • |
| GO Bio Pro | cellular response to nerve growth factor stimulus |  |  |  |  |  |  | • |
| Reactome | TRIF(TICAM1)-mediated TLR4 signaling |  |  |  |  |  |  | • |
| WikiPathways | Insulin Signaling |  |  |  |  |  |  | • |
| GO Bio Pro | intracellular receptor signaling pathway |  |  |  |  |  |  | • |
| GO Bio Pro | antigen receptor-mediated signaling pathway |  |  |  |  |  |  | • |
| GO Mol Func | steroid hormone receptor binding |  |  |  |  |  |  | • |
| Reactome | FRS-mediated FGFR3 signaling |  |  |  |  |  |  | • |
| KEGG | PI3K-Akt signaling pathway |  |  |  |  |  |  | • |

Supplementary Table 3. Pathway Groups (cont.)

| Database | Pathway | Cancer | Motility | DNA Damage | Cell Cycle | Apoptosis | Miscellaneous | Canonical |
| --- | --- | --- | --- | --- | --- | --- | --- | --- |
| GO Bio Pro | regulation of JNK cascade |  |  |  |  |  |  | • |
| WikiPathways | IL-7 Signaling Pathway |  |  |  |  |  |  | • |
| Reactome | MyD88-independent TLR4 cascade |  |  |  |  |  |  | • |
| WikiPathways | p38 MAPK Signaling Pathway |  |  |  |  |  |  | • |
| Reactome | Signaling by Nuclear Receptors |  |  |  |  |  |  | • |
| WikiPathways | TNF related weak inducer of apoptosis (TWEAK) Signaling Pathway |  |  |  |  |  |  | • |
| GO Bio Pro | regulation of peroxisome proliferator activated receptor signaling pathway |  |  |  |  |  |  | • |
| GO Bio Pro | negative regulation of intracellular steroid hormone receptor signaling pathway |  |  |  |  |  |  | • |
| GO Bio Pro | canonical Wnt signaling pathway |  |  |  |  |  |  | • |
| Reactome | Signaling by NOTCH3 |  |  |  |  |  |  | • |
| Reactome | Signaling by FGFR4 |  |  |  |  |  |  | • |
| Reactome | Signaling by Insulin receptor |  |  |  |  |  |  | • |
| KEGG | mTOR signaling pathway |  |  |  |  |  |  | • |
| WikiPathways | EGF/EGFR Signaling Pathway |  |  |  |  |  |  | • |
| Reactome | Interleukin-12 family signaling |  |  |  |  |  |  | • |

Supplementary Table 4. Criteria for Pathway Group Classification

| Pathway group | Inclusion criteria | Exclusion criteria |
| --- | --- | --- |
| Cancer | “cancer”, “glio” | Inclusion in Canonical pathway group; Specific to non-glioma tumors |
| Motility | “actin”,”adhe”, “chemotaxis”, “cytoskeleton”, “ECM”, “filopodi”, “integrin”, “lamellipo”, “leading edge”, “locomotion”, “microtubule”, “migration”, “movement”, “polarity”, “projection”, “size” | Inclusion in Canonical pathway group; Specific to unrelated cell type |
| DNA | “DNA damage”, “repair” | “apopto” |
| Cell Cycle | “cell growth”, “cycle”, “mito”, “number”, “replication” | “developmental”, “metabolic”, “mitochondrial”, “ovulation”, “vesicle”, “viral”; Inclusion in Canonical pathway group; Specific to unrelated cell type |
| Apoptosis | “apopto” | Inclusion in Canonical pathway group; Specific to unrelated cell type |
| Miscellaneous | “mechanical”, “miRNA”, “RISC” | Inclusion in other pathway groups |
| Canonical | “cascade”, “effector”, “event”, “receptor”; specific ligand/receptor and either “activity” or “growth factor” or “network” or “pathway” or “signaling” | “apopto”, “cyclin”, “enzyme”, “mutant”, “nanoparticle”, “non-canonical”, “phagocytosis”, “therapy-induced”, “vesicle”, “variant”; Specific to unrelated cell type |
